# MicroRNA-based diagnostic tools for advanced fibrosis and cirrhosis in patients with chronic hepatitis B and C

**DOI:** 10.1038/srep34935

**Published:** 2016-10-12

**Authors:** Kevin Appourchaux, Safi Dokmak, Matthieu Resche-Rigon, Xavier Treton, Martine Lapalus, Charles-Henry Gattolliat, Emmanuelle Porchet, Michelle Martinot-Peignoux, Nathalie Boyer, Michel Vidaud, Pierre Bedossa, Patrick Marcellin, Ivan Bièche, Emilie Estrabaud, Tarik Asselah

**Affiliations:** 1 INSERM, UMR1149, Team «Physiopathologie et traitements des hépatites virales», Centre de Recherche sur l’Inflammation, and Université Denis Diderot Paris 7, site Bichat, BP 416, F-75018, Paris, France; 2 Service d’hépatologie, PMAD Hôpital Beaujon, 100 Bd du Général Leclerc, Clichy la Garenne, 92110 Clichy Cedex, France.; 3 Laboratory of Excellence Labex INFLAMEX, PRES Paris Sorbonne Cité, Paris, France; 4 Department of HPB Surgery and Liver Transplantation, Beaujon Hospital, Paris, France; Assistance Publique Hôpitaux de Paris, Paris, France; Université Paris VII Denis Diderot, Paris, France; 5 Service de Biostatistique et Information Médicale, Hôpital Saint-Louis, AP-HP, ECSTRA Team, Inserm UMR1153, Paris, France; 6 Service de gastroentérologie, MICI et Assistance Nutritive, Hôpital Beaujon, Assistance Publique Hôpitaux de Paris, Clichy, France; 7 UMR745 INSERM, Université Paris Descartes, Sorbonne Paris Cité, Faculté des Sciences Pharmaceutiques et Biologiques, Paris, France; 8 Service d’anatomopathologie, Hôpital Beaujon, Assistance Publique Hôpitaux de Paris, Clichy, France

## Abstract

Staging fibrosis is crucial for the prognosis and to determine the rapid need of treatment in patients with chronic hepatitis B (CHB) and C (CHC). The expression of 13 fibrosis-related microRNAs (miRNAs) (miR-20a, miR-21, miR-27a, miR-27b, miR-29a, miR-29c, miR-92a, miR-122, miR-146a, miR-155, miR-221, miR-222, and miR-224) was analyzed in 194 serums and 177 liver biopsies of patients with either CHB or CHC to develop models to diagnose advanced fibrosis and cirrhosis (Metavir F3-F4). In CHB patients, the model (serum miR-122, serum miR-222, platelet count and alkaline phosphatase) was more accurate than APRI and FIB-4 to discriminate in between mild and moderate fibrosis (F1-F2) and F3-F4 (AUC of CHB model: 0.85 vs APRI: 0.70 and FIB-4: 0.81). In CHC patients, the model (hepatic miR-122, hepatic miR-224, platelet count, albumin and alanine aminotransferase) was more accurate than both APRI and FIB-4 to discriminate in between patients with F3-F4 and F1-F2 (AUC of the CHC model = 0.93 vs APRI: 0.86 and FIB-4: 0.79). Most of the miRNAs tested were differentially expressed in patients with CHB and CHC. In particular, serum miR-122 was 28-fold higher in patients with CHB than in those with CHC. Both CHB and CHC models may help for the diagnosis of advanced fibrosis and cirrhosis (F3-F4).

Chronic hepatitis B (CHB) and C (CHC) affect respectively 350 and 170 million individuals worldwide^1^. Patients with CHB and CHC are at high risk to develop liver fibrosis, cirrhosis and hepatocellular carcinoma (HCC)^2^. The evaluation of liver fibrosis is crucial for assessing the prognosis and the need of treatment^3^,^4^,^5^,^6^. Scoring systems (Knodell, Metavir, Ishak, etc.) provide a semi-quantitative assessment for individual clinical prognosis, cross-sectional, cohort studies and treatment trial. In patients with CHB and CHC, the liver biopsy remains the gold standard to assess fibrosis despite inter-/intra-observer variations and sampling errors^7^.

Alternate non-invasive methods have been initially developed and evaluated in patients with CHC^8^ and increasingly evaluated in CHB^9^,^10^. They include transient elastography and serological scores such as the “aspartate aminotransferase (AST)-to-platelet ratio index” (APRI)^11^ and the “four factors-based fibrosis index” (FIB-4)^12^. Both index have a moderate sensitivity and accuracy to discriminate in between each of the stages of fibrosis of the Metavir score^13^,^14^. While the World Health Organization (WHO) recommends the use of APRI in resource-limited countries^15^,^16^, a recent study suggested that APRI and FIB-4 are not suitable enough for use in clinical practice in CHB patients^10^. Whereas serological scores are very suitable to detect the absence of fibrosis or cirrhosis and severe fibrosis, their performance remains low to diagnose moderate fibrosis (Metavir F2)^8^.

MicroRNAs (miRNAs) are small non-coding endogenous RNAs that regulate up to 60% of the expression of cellular mRNAs. Previous studies showed that several miRNAs regulate liver functions and are associated with HCC^17^. Moreover, different sets of miRNAs have been associated with CHB and CHC-induced fibrosis and inflammatory liver diseases^17^,^18^. Nevertheless, those studies mainly focused on cirrhosis. Little is known about the level of miRNAs expression within intermediate stages of fibrosis.

MiR-122 enhances the replication of HCV^19^. We previously reported a reduction of the hepatic expression of miR-122 in patients with CHC at the most advanced stages of fibrosis^20^. MiR-122 has been suggested to interact with HBV genome and to inhibit its replication^21^. Interestingly, in patients with CHB, the down-regulation of miR-122 may contribute to viral persistence and carcinogenesis^22^.

The main objective of the study was to identify miRNAs associated with fibrosis in order to establish miRNAs-based models to discriminate between patients with advanced fibrosis and cirrhosis (F3-F4) and those with mild to moderate fibrosis (F1-F2) in CHB and CHC. The expression profiles of 13 selected miRNAs were analyzed in serum and liver biopsies of patients with CHB and CHC. Models to diagnose severe fibrosis and cirrhosis (F3-F4) in CHC and CHB were established based on multivariate analysis. Secondary objectives of this work were to study the correlation between the expression of miRNAs in serum and liver.

## Results

### Patients and baseline characteristics

In the total cohort (CHB and CHC patients), 142 patients with mild (F1) to moderate (F2) fibrosis (F1-F2) and 138 patients with advanced fibrosis (F3) to cirrhosis (F4) (F3-F4) were included (Table S1). The mean age was 47 years in both patients with F1-F2 and those with F3- F4 (F3-F4). Patients with F1-F2 had lower body mass index (BMI) (p = 0.034), alanine amino transferase (ALT) (p = 0.0002), aspartate amino transferase (AST) (p < 0.0001), alkaline phosphatase (APL) (p < 0.0001), gamma glutamyltransferase (GGT) (p < 0.0001), total bilirubin (p = 0.005), glycemia (p = 0.0002) and cholesterol (p < 0.0001) than patients with F3-F4 (Table S1).

Patients with CHC were older (p < 0.0001), had higher AST (p = 0.025), ALP (p = 0.0003), GGT (p < 0.0001), glycemia (p = 0.01), platelet count (p = 0.04) and prothrombin time (PT) (p < 0.0001) than patients with CHB (Table S2).

In the CHB cohort, 58 and 44 patients with respectively F1-F2 and F3-F4 were included. Thirty-nine out of the 103 patients were HBe antigen positive (data not shown). CHB patients with F1-F2 had lower AST (p = 0.047), ALP (p = 0.0002), GGT (p = 0.001), glycemia (p = 0.027) and triglycerides (p = 0.035) than CHB patients with F3-F4. CHB patients with F1-F2 had higher platelet count (p = 0.003), PT (p = 0.011) and albumin (p = 0.002) than CHB patients with F3-F4 (Table 1).

In the CHC cohort, 84 patients with F1-F2 and 94 with F3-F4 were enrolled (Table 1). The mean age at liver biopsy was 49 years. All genotypes were represented with 51.95%, 14.29%, 14.29%, 14.29%, 3.90% and 1.30% of patients with respectively genotypes 1, 2, 3, 4, 5 and 6. CHC patients with F1-F2 had lower ALT (p = 0.0002), AST (p < 0.0001), ALP (p < 0.0001), GGT (p < 0.0001), total bilirubin (p = 0.011), glycemia, (p = 0.0003) and cholesterol (p < 0.0001) than CHC patients with F3-F4 (Table 1). CHC patients with F1-F2 had higher platelet count (p < 0.0001), cholesterol (p < 0.0001), and PT (p = 0.002) than those with F3-F4 (Table 1).

### Differential hepatic miRNAs expression in patients with advanced fibrosis and cirrhosis

**Figure 1:**
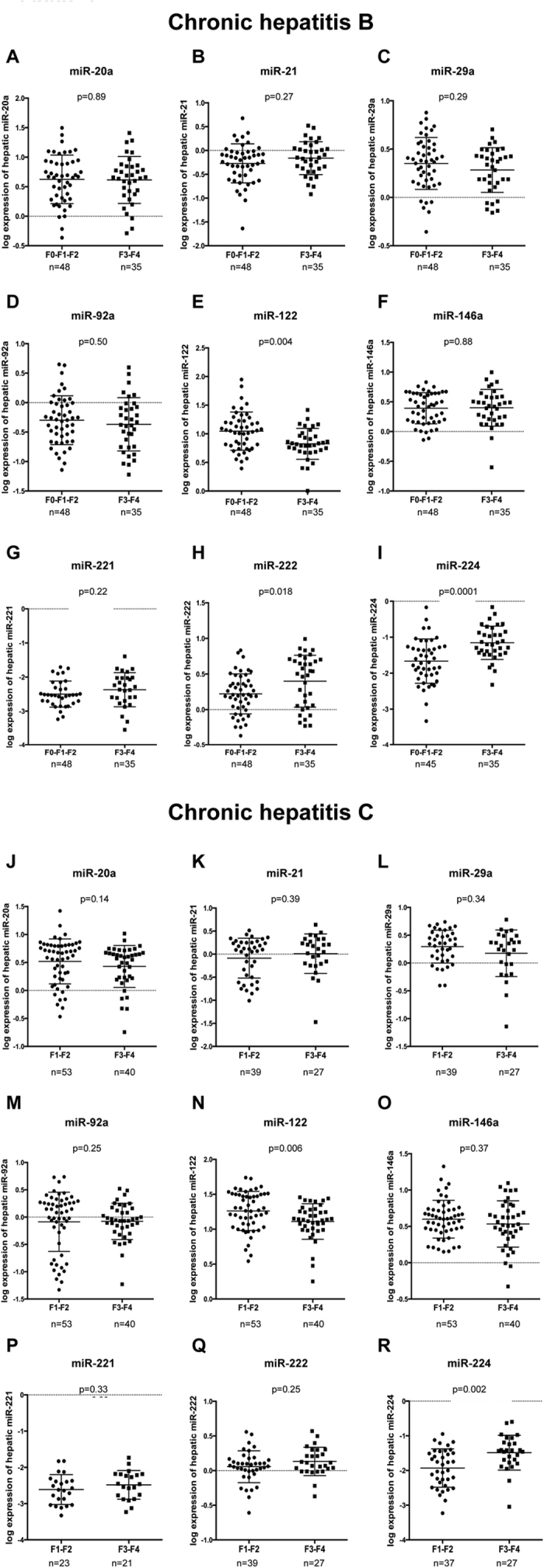
Differences in the expression of hepatic miRNAs according to the stage of fibrosis in patients with chronic hepatitis B and C. The expression of hepatic miR-20a, miR-21, miR-29a, miR-92a, miR-122, miR-146a, miR-221, miR-222, miR-224 was assessed by RT-qPCR from 1 ng of cDNA and compared in patients with F3-F4 and F0-F1-F2, in chronic hepatitis B patients (**A–I**) and in those with chronic hepatitis C (**J–R**). The ΔCt (ΔCt = 2^ΔCp,sample^) of each miRNA was calculated and normalized to the ΔCt value of SNORD44 in each biopsy. The log expression of the ratio miRNA/SNORD44 is shown as dot plot, each dot represents one patient (mean and standard deviation). The Wilcoxon rank-sum test was used to compare miRNAs expression.

The hepatic expression of 13 miRNAs (miR-20a, miR-21, miR-27a, miR-27b, miR-29a, miR-29c, miR-92a, miR-122, miR-146a, miR-155, miR-221, miR-222, and miR-224) was compared between patients with F3-F4 and those with F1-F2 either in patients with CHB or CHC (Fig. 1 and Supplementary Figure 1).

In patients with CHB, hepatic miR-122 (p = 0.004) (Fig. 1E) and miR-27b (p = 0.038) (Supplementary Figure 1B) were decreased in patients with F3-F4 compared to those with F1-F2. Both miR-222 (p = 0.018) (Fig. 1H) and miR-224 (p = 0.0001) (Fig. 1I) were increased in patients with F3-F4 compared to those with F1-F2 (Fig. 1).

In patients with CHC, hepatic miR-122 was reduced in patients with F3-F4 compared to those with F1-F2 (p = 0.008) (Fig. 1N) and hepatic miR-224 was increased in patients with F3-F4 compared to those with F1-F2 (p = 0.002) (Fig. 1R). The expression of hepatic miR-222 was not significantly different between patients with F3-F4 and those with F1-F2 (Fig. 1I).

Hepatic miR-20a, miR-21, miR-29a, miR-92a, miR-146a, miR-221 (Fig. 1A–D,F,J–M,O), miR-27a, miR-29c and miR-155 (Supplementary Figure 1A, 1C-1H), showed no significant differences of expression between patients with F3-F4 and F1-F2 with either CHB or CHC.

### Differential serum miRNAs expression in patients with advanced fibrosis and cirrhosis

**Figure 2:**
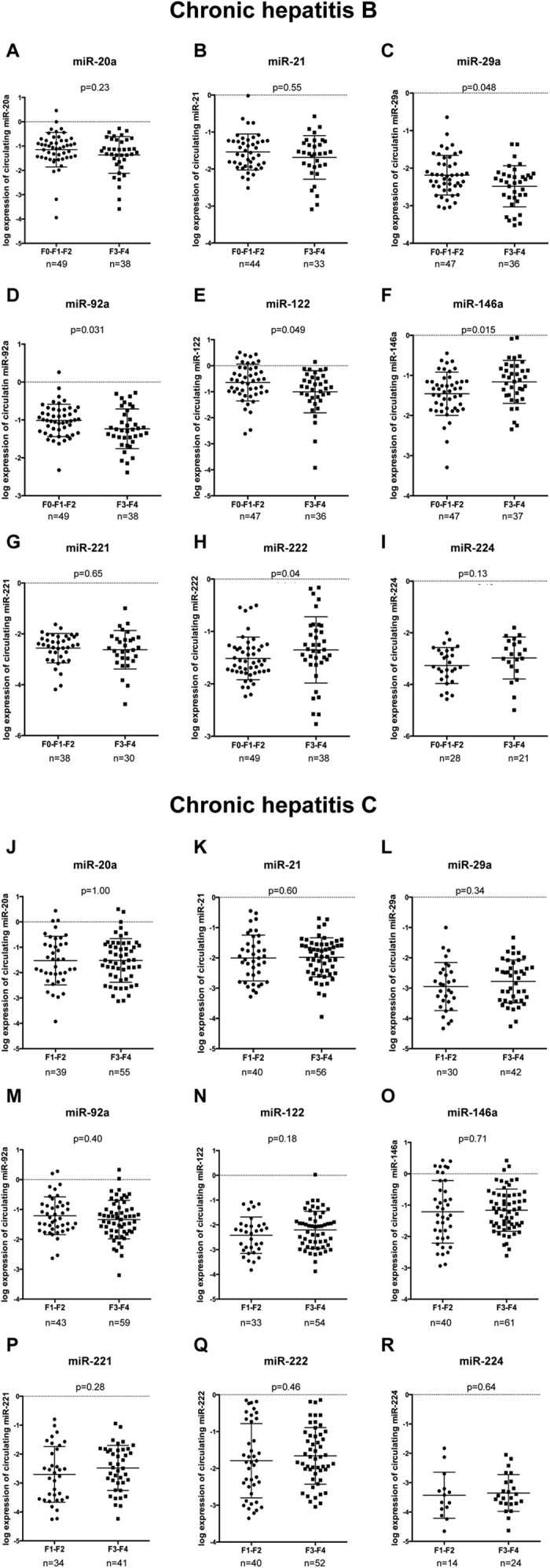
Differences of the expression of serum miRNAs according to the stages of fibrosis in patients with chronic hepatitis B and C. The expression of serum miR-20a, miR-21, miR-29a, miR-92a, miR-122, miR-146a, miR-221, miR-222, miR-224 was assessed by RT-qPCR and compared in patients with F3-F4 and F0-F1-F2, in chronic hepatitis B patients (**A–I**) and in those with chronic hepatitis C (**J–R**). The ΔCt (ΔCt = 2^ΔCp,sample^) of each miRNA was calculated and normalized to the ΔCt value of the exogenous *C. elegans*-miR-39 (*cel*-miR-39) in each serum. The log expression of the ratio miRNA/*cel*-miR-39 is shown as dot plot, each dot represents one patient (mean and standard deviation). The Wilcoxon rank-sum test was used to compare miRNAs expression.

The serum expression of 13 miRNAs (miR-20a, miR-21, miR-27a, miR-27b, miR-29a, miR-29c, miR-92a, miR-122, miR-146a, miR-155, miR-221, miR-222, and miR-224) was compared to patients with CHB and CHC with F3-F4 and in those with F1-F2 (Fig. 2 and Supplementary Figure 2).

In patients with CHB, the serum expression of miR-29a (p = 0.048), (Fig. 2C) miR-92a (p = 0.031) (Fig. 2D), and miR-122 (p = 0.049) (Fig. 2E) was reduced in patients with F3-F4 compared to those with F1-F2. Moreover, the expression of miR-146a (p = 0.015) (Fig. 2F) and miR-222 (Fig. 2H) (p = 0.040) was increased in patients with F3-F4 compared to those with F1-F2. Serum expression of miR-20a, miR-21, miR-221 andmiR-224, miR-27a, miR-27b, miR-29c, miR-155 (Fig. 2A,B,G,Q, and Supplementary Figure 2A–D) showed no significant differences of expression between CHB patients with F3-F4 and those with F1-F2.

MiR-29a, -92a, -122, -146a and -222 were all deregulated in patients with F3-F4 compared to those with F0-F2. Therefore the expression of these 5 miRNAs was tested in a group of seven patients with CHB who started nucleosides/nucleotides inhibitors (Nucs) therapy to abrogate HBV replication. One serum was available before the initiation of treatment and a second one 12.5 months (IQ: 11.5-14) after the initiation of Nucs (Table S3). In agreement with expectations^23^, serum HBV DNA was undetectable at the time of the second serum for the 7 patients (Table S3). None of the miRNAs tested (eg. miR-29a, -92a, -122, -146a and -222) was differentially expressed after abrogation of HBV replication following one year of Nucs therapy (Supplementary Figure 3A–E).

In patients with CHC, the serum expression of miR-20a, miR-21, miR-29a, miR-92a, miR-122, miR-146a, miR-221, miR-222, and miR-224 (Fig. 2J–R) showed no difference in patients with F3-F4 when compared to those with F1-F2 (Fig. 2). The level of expression of serum miR-27a, miR-27b, miR-29c and miR-155 was under the limit of detection in patients with CHC (data not shown). In CHC patients since none of the miRNAs tested was significantly deregulated in serum, we followed the expression of the miRNAs that were deregulated in the liver and/or those almost significantly differentially expressed in serum samples of patients with F3-F4 compared to those with F0-F1-F2. (eg. MiR-122, -221, -222 and -224) in 9 patients before and after sustained virological response following IFN-based treatment (n = 8) or DAAs (n = 1). Sustained virological response is defined as HCV RNA undetectable 12 weeks after the end of treatment^24^. For these 9 patients the second serum was available 12.0 (IQ: 9–13) months after the end of the treatment (Table S3). None of these miRNAs was differentially expressed after HCV eradication (Supplementary Figure 3G–I). However, a more detailed analysis of fibrosis-associated miRNAs in treated CHB and CHC patients may inform on the recovery and/or regression of fibrosis while avoiding liver biopsy.

### MiRNAs-based signature in patients with CHB and comparison of its diagnostic performance with APRI and FIB-4

In univariate analysis, all the following variables (aspartate aminotransferase (AST), alanine aminotransferase (ALT), alkaline phosphate (ALP), albumin, platelet count, serum miR-29a, miR-92a, miR-122, miR-146a and miR-222) were associated with F3-F4 (Fig. 2). The characteristics of the patients for whom serum samples were available were used for the establishment of the CHB model (Table S4). Serum miR-222 (OR = 1.12; 95% CI: 1.01–1.24; p = 0.03) and ALP OR = 1.21; 95% CI: 1.04–1.41; p = 0.02) levels were independently associated with stages of fibrosis (Table 2). Combined together, serum miR-122, serum miR-222, ALP and platelet count were the strongest associated predictors of advanced fibrosis and cirrhosis (F3-F4) (Table 2). ROC analysis was performed to evaluate the diagnostic performances of the model combining serum miR-122, serum miR-222, platelet count and ALP levels to discriminate patients with F3-F4 from those with F1-F2.

The model combining serum miR-122, serum miR-222, platelet count and ALP (called CHB model) discriminates patients with F3-F4 from those with F1-F2 with an AUC of 0.86 (95% CI = 0.77–0.95) (Table 3). This CHB model was more accurate than FIB-4 (AUC = 0.81; 95% CI = 0.71–0.90) and APRI (AUC = 0.70; 95% CI = 0.58–0.82) to discriminate patients with F3-F4 from those with F1-F2 (Table 3). The CHB model was statistically better than APRI (p = 0.007) but not than FIB-4 (p = 0.39) (Table 3). By optimizing with the Youden’s index, the optimal cut-off of the CHB model for the diagnosis of patients with F3-F4 was: 0.57 (sensitivity (Se) = 0.79, specificity (Sp) = 0.95, PPV = 0.79, NPV = 0.95) (Table 4).

To optimally discriminate between patients with F3-F4 and those with F1-F2 both APRI and FIB-4 need 2 different cut-offs (<0.5 and >1.5 for APRI and <1.45 and >3.25 for FIB-4).

Taking into account the cut-offs to differentiate patients with advanced fibrosis and cirrhosis (F3-F4) from patients with mild-moderate fibrosis (F1-F2), the sensitivity of the CHB model (Se = 0.79) was respectively higher and lower than the one of FIB-4 (Se = 0.67) and the one of APRI (Se = 0.84) (Table 4). Moreover, the specificity of our CHB model (Sp = 0.95) was higher than the one of APRI (Sp = 0.90) and lower than the one of FIB-4 (Sp = 0.97) (Table 4).

The positive predictive value (PPV) of the CHB model (PPV = 0.79) was higher than the one of APRI (PPV = 0.27 for the 0.5 cut-off and PPV = 0.46 for the 3.25 cut-off) and FIB-4 (PPV = 0.38 for the 1.45 cut-off and 0.65 for 1.5 cut-off) (Table 4).

The negative predictive value (NPV) of our CHB model (NPV = 0.95) was higher than the ones of FIB-4 (NPV = 0.90 for the 1.45 cut-off and 0.84 for 3.25 cut-off) and APRI (NPR = 0.91 for the 0.5 cut-off and 0.85 for the 1.5 cut-off) (Table 4).

Respectively 32 (14 + 18) and 28 (7 + 21) patients of the 44 patients with F3-F4 had a FIB-4 < 3.25 and an APRI < 1.5 that may account for relatively low PPV of APRI and FIB-4 in our cohort of patients (Tables S5 and S6).

### MiRNAs-based signature in patients with CHC and comparison of its diagnostic performance with APRI and FIB-4

In univariate analysis, albumin, platelet count, AST, ALT, ALP, hepatic miR-122 and miR-224 were all associated with F3-F4. Therefore, these variables were considered for the model selection of the multivariate analysis. The characteristics of the patients for whom liver samples were available were used for the establishment of the CHC model (Table S7). The strongest independently associated predictors of F3-F4 were hepatic miR-122 (p = 0.01), hepatic miR-224 (p = 0.03) and platelets count (p = 0.004) (Table 2). ROC analysis was performed to evaluate the diagnostic performances of the model to discriminate patients with F3-F4 from those with F1-F2.

The model combining hepatic miR-122, hepatic miR-224, platelets count, ALT and albumin (defined as the CHC model) was the strongest one to discriminate patients with F3-F4 from those with F1-F2 with an AUC of 0.93 (95%, CI = 0.83–0.87). The CHC model was more accurate than both FIB-4 (AUC = 0.79, 95% CI = 0.67–0.90) and APRI (AUC = 0.86, 95% CI = 0.75–0.96) to discriminate F3-F4 from F1-F2 (Table 3). The CHC model was statistically better than FIB-4 (p = 0.009) to discriminate patients with F3-F4 from those with F1-F2 (Table 3).

As for the CHB model, a single cut-off of 0.32 was defined for the CHC model by optimizing with the Youden’s index (Table 4). Therefore, there was no grey zone in the calculation of CHB and CHC value and the fibrosis was predictable for all the patients.

Taking into account the two different cut-offs to differentiate patients with F3-F4 from those with F1-F2 for both APRI and FIB-4, the sensitivity of the CHC model (0.89) was higher than the one of FIB-4 (Se = 0.76) and lower than the one of APRI (Se = 0.91) (Table 4).

The specificity of the CHC model (Sp = 0.84) was lower than the ones of FIB-4 (Sp = 0.99 with the cut-off of 3.25) and APRI (0.95 with the cut-off of 1.5) (Table 4).

The PPV of our CHC model (PPV = 0.58) was lower than the ones of APRI (PPV = 0.84 for the cut-off of 3.25) and FIB-4 (0.65 for the cut-off of 1.5) (Table 4).

The NPV of the CHC model (NPV = 0.97) was higher than the ones of APRI (0.95 for the cut-off of 0.5 and 0.86 for cut-off of 1.5) and FIB-4 (0.92 for the cut-off of 0.5 and 0.84 for the cut-off of 1.5) (Table 13) irrespective of the cut-off values. Respectively 58 (19 + 39) and 50 (7 + 43) patients of 94 patients with F3-F4 had a FIB-4 < 3.25 and an APRI < 0.5 that may account for relatively low PPV of APRI and FIB-4 in our cohort of patients (Tables S5 and S6).

### Differential miRNAs expression between patients with CHB and CHC

**Figure 3:**
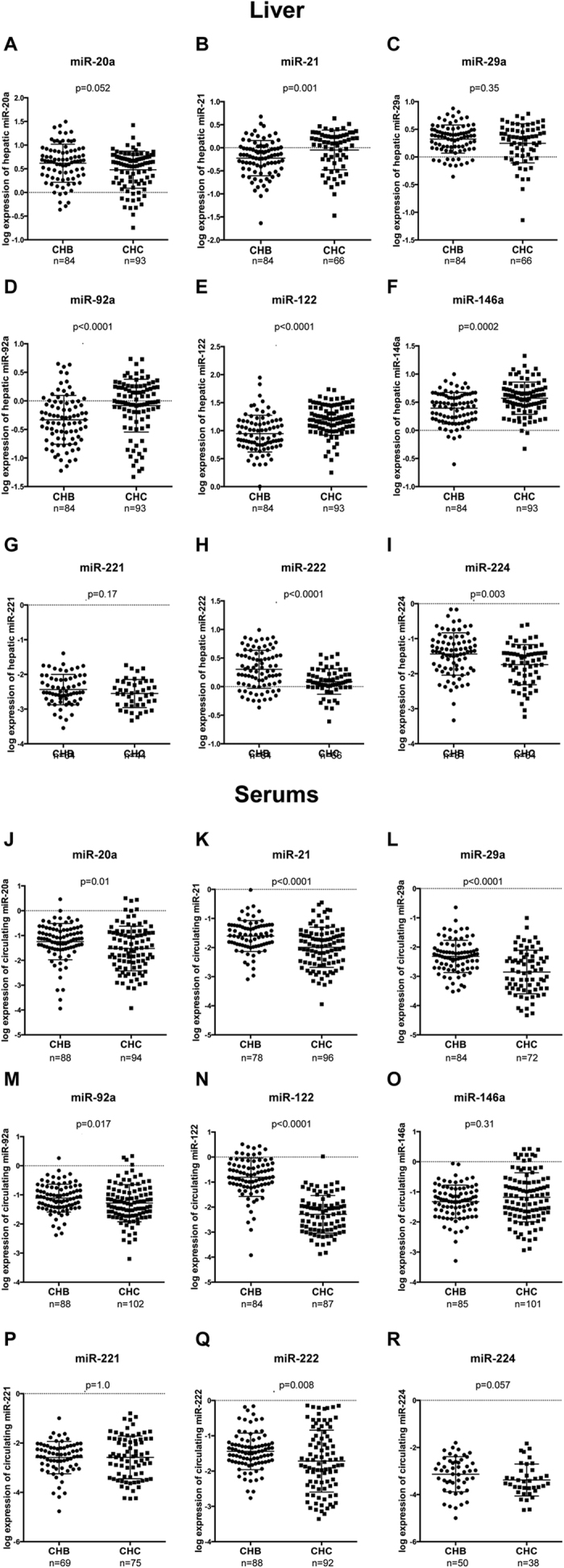
Comparison of the expression of serum and hepatic miRNAs in patients with chronic hepatitis B and C. The expression of serum (**A–I**) and hepatic (**J–R**) expression of miR-20a, miR-21, miR-29a, miR-92a, miR-122, miR-146a, miR-221, miR-222 and miR-224 was assessed by RT-qPCR and compared in patients with CHC and CHB. (**A–I**) In serum samples, The ΔCt (ΔCt = 2^ΔCp,sample^) of each miRNA was calculated and normalized to the ΔCt value of the exogenous *C. elegans*-miR-39 (*cel*-miR-39) in each serum. The log expression of the ratio miRNA/*cel*-miR-39 is shown as dot plot, each dot represents one patient (mean and standard deviation). (**J–R**) In liver samples, the ΔCt (ΔCt = 2^ΔCp,sample^) of each miRNA was calculated and normalized to the ΔCt value of SNORD44 in each biopsy. The log expression of the ratio miRNA/SNORD44 is shown as dot plot, each dot represents one patient (mean and standard deviation). The log expression of the ratio miRNA/SNORD44 is shown as dot plot, each dot represents one patient (mean and standard deviation). The Wilcoxon rank-sum test was used to compare miRNAs expression.

Hepatic miR-20a (p = 0.052), miR-29a (p = 0.35), miR-29c (p = 0.17) and miR-221 (p = 0.17) were expressed in an equivalent way in the liver of patients with CHC and CHB (Figure). However, hepatic miR-21 (p = 0.001), miR-92a (p < 0.0001), miR-122 (p < 0.0001), miR-146a (p = 0.0002), miR-27a (p < 0.0001), miR-27b (p = 0.0002) and miR-155 (p < 0.0001) were increased in the liver of patients with CHC as compared to those with CHB (Fig. 3B,D–F and Supplementary Figure 4A,B,D). Interestingly, the level of expression of hepatic miR-122 in patients with CHC was 1.96-fold higher than in patients with CHB, independently of the stage of fibrosis (Fig. 3). Hepatic miR-222 and miR-224 were decreased in patients with CHC compared to those with CHB.

Serum miR-146a (p = 0.31), miR-221 (p = 0.35), and miR-224 (p = 0.057) were expressed in an equivalent way in the serum of patients with CHB and CHC (Fig. 3). However, serum miR-20a (p = 0.010), miR-21 (p < 0.0001), miR-29a (p < 0.0001), miR-92a (p = 0.017) miR-122 (p < 0.0001) and miR-222 (p = 0.008) were decreased in serums of patients with CHC compared to those with CHB (Fig. 3). Interestingly, the level of expression of serum miR-122 was 28-fold higher in patients with CHB than in those with CHC, independently of the stage of fibrosis.

### Correlations between serum and hepatic miRNAs expression

In order to assess if serum miRNAs levels can reflect the level of hepatic miRNAs, we analyzed the correlation between the level of serum miRNAs and the level of hepatic miRNAs in patients with CHB and CHC.

Hepatic and serum miR-20a (R^2^ = 0.31, p = 0.01) and miR-146a (R^2^ = 0.36, p = 0.009) expression were correlated. No correlation was observed for other miRNAs (Table S8). An inversed correlation was observed between hepatic miR-20a (R^2^ = −0.31; p-value = 0.01), miR-92a (R^2^ = −0.25; p-value = 0.02), and miR-122 (R^2^ = −0.25; p-value = 0.01) and ALT (Table S9). An inversed correlation was observed between hepatic miR-20a (R^2^ = −0.36; p-value = 0.01), miR-92a (R^2^ = −0.27; p-value = 0.01), and miR-122 (R^2^ = −0.35; p-value = 0.01), and AST (Table S9). No correlation was observed between the other studied miRNAs and AST and ALT (Data not shown).

### Correlations between the expression of miRNAs and ALT and AST

In the CHB cohort, serum miR-122 was correlated with ALT (R^2^ = 0.28, p = 0.01) and serum miR-21 was positively correlated with both ALT (R^2^ = 0.23, p = 0.04) and AST (R^2^ = 0.23, p = 0.04) (Table 5).

In the CHC cohort, serum miR-122 was positively correlated with the levels of both ALT (R^2^ = 0.28, p = 0.01) and AST (R^2^ = 0.31, p = 0.009). No significant correlations were reported between the expression of the others serum miRNAs and the level of ALT and AST (Table 5).

## Discussion

The major novelty of our work consists in the identification of two models based on the expression of miRNAs designed to diagnose advanced fibrosis and cirrhosis (F3-F4) in patients with CHB and CHC. To develop these models, we report differential profiles of expression of miRNAs in the liver and serum of patients with CHB and CHC.

The non-invasive model combining serum miR-122, serum miR-222, platelet count and alkaline phosphatase (ALP) for CHB patients allowed the identification of patients with F3-F4 from those with F1-F2. Interestingly, the CHB model had higher specificity than APRI (0.95 vs 0.90) and a higher sensitivity than FIB-4 (0.79 vs 0.67) (Table 4). Moreover with a single cut-off, this model was suitable to identify advanced fibrosis and cirrhosis (F3-F4) in all the patients tested. Overall, the CHB model was more accurate than APRI and FIB-4 to stage fibrosis (F3-F4 vs F1-F2) in all patients with CHB (Se = 0.79, Sp = 0.95, PPV = 0.79, NPV = 0.95) (Table 4). Whereas, the CHB model may help to monitor fibrosis for both positive and negative HBeAg carriers, it should be validated in a larger cohort of patients to be used in clinical practice.

The reduction of hepatic and serum miR-122 in CHB patients (Figs 1 and 2) with F3-F4 is in agreement with previous reports^18^,^20^. Serum miR-122 is a relevant biomarker in liver diseases^25^,^26^. Indeed, the assessment of serum miR-122, miR-21 and miR-29c discriminates patients with cirrhosis (F4) among patients with CHB^18^.

The expression of both serum and hepatic miR-222 was increased in CHB patients with F3-F4 compared to those with F1-F2 (Figs 1 and 2). MiR-222 promotes the proliferation and differenciation of hepatic stellate cells (HSCs)^27^. Moreover, miR-222 downregulates the expression of ICAM-1^28^ which is responsible for the transmigration of immune cells to the sites of liver damage^29^. Interestingly, the up-regulation of miR-222 in F3-F4 patients may accelerate the activation of HSCs, increases the inflammation in the liver and thus facilitates the progression of fibrosis.

Although the World Health Organization recommended the use of APRI to detect significant fibrosis and cirrhosis (F2-F3-F4) in resource-limited countries^15^,^16^ its accuracy and sensitivity remain moderate to discriminate in between the stages of fibrosis in patients with CHB^10^,^14^. FIB-4 is suggested to be superior to APRI to diagnose cirrhosis in patients with CHB^30^,^31^,^32^. However, overall because of the weakness of their diagnostic performance, APRI and FIB-4 do not seem to be suitable for use in clinical practice in patients with CHB^10^,^14^. Other non-invasive tests have been developed to stage fibrosis in patients with CHB. The combination of AST, GGT, AFP and platelet count was able to diagnose severe fibrosis and cirrhosis (F3-F4) in CHB patients with an AUC of 0.85^33^. The Platelet count/Age/ALP/Alpha-foeto-protein/AST (PAPAS) index was able to diagnose significant fibrosis (F2-F3-F4) with an AUC of 0.776 and a NPV of 88.4%^34^. Interestingly, these two last scores take both into account the level of AST that is not always associated with histological lesions in CHB patients^35^. Likewise PAPAS index, the non-invasive miRNAs-based model for patients with CHB includes ALP and platelet count, suggesting that those two biomarkers are particularly relevant to diagnose fibrosis. More recently, the model including GGT to platelet ratio (GPR) developed in patients with CHB was less accurate than APRI and FIB-4 to identify significant fibrosis (F2-F3-F4) and cirrhosis^36^.

The CHC model combining hepatic miR-122, hepatic miR-224, platelet count, ALT and albumin allowed the identification of CHC patients with F3-F4 from those with F1-F2 with better diagnostic performances than APRI and FIB-4 (AUC = 0.93, 95% CI = 0.87–0.99, Se = 0.89, Sp = 0.84, NPV = 0.97) (Table 4). Since this model takes into account hepatic expression of miR-122 and miR-224, its use may be limited compared to APRI and FIB-4. However, a risk of miss-classification of 20% is associated with liver histology staging^7^,^37^,^38^. Therefore, the quantification of hepatic miR-122 and miR-224 in patients with CHC who undergo liver biopsy may help to better characterize the stage of fibrosis and reduce the risk of mis-classification.

Several studies described additional analyses such as image analysis and liver gene expression assessment that may help to diagnose more precisely the stages of fibrosis from liver biopsy^39^,^40^,^41^. Moreover, the identification of genes and miRNAs which expression is modified at different stages of fibrosis is relevant to elucidate its molecular mechanisms.

Whereas the progression of fibrosis is a common feature of both CHB and CHC, it is likely that the molecular mechanisms of fibrosis may be specific to each type of virus. Indeed, some miRNAs were differentially expressed the same way in F3-F4 vs F1-F2 patients with CHC and CHB and some miRNAs showed strong variations in between patients with CHB and in those with CHC independently of the stage of fibrosis (Fig. 3).

Our results suggest that the reduction of hepatic miR-122 and the increase of hepatic miR-224 may be a common feature of CHB and CHC-induced fibrosis (Figs 1 and 2). We, and others, already reported the reduction of hepatic miR-122 in patients with CHC, at later stages of fibrosis^20^,^42^. It might be suggested that the pool of functional hepatocytes decreases at the most advanced stages of fibrosis (F3-F4). Since the expression of hepatic miR-122 is assessed from biopsy of the whole liver, the reduction of miR-122 expression, we observed, may be a consequence of the reduction of the number of hepatocytes. Indeed, miR-122 is mainly expressed in hepatocytes whereas SNORD44 which is used for normalization is expressed at the same level in the different types of hepatic cells. Another explanation for the reduction of hepatic miR-122 in patients with F3-F4 may be the activation of HSCs and viral infection that were both associated with a decrease of miR-122 (reviewed in ref. 25).

Upregulation of hepatic miR-224 has been described in various types of cancer including HCC^43^,^44^. Thereby, the increased hepatic expression of miR-224 in patients with F3-F4 (Fig. 1) might emphasize that those patients are more likely to develop HCC compared to patients with F1-F2 who show lower level of hepatic miR-224. Interestingly, a previous report suggested that serum miR-224 was a reliable biomarker for the detection of early-stage of HCC^45^. This common characteristic observed in both CHB and CHC patients might represent interesting targets for anti-fibrotic therapies.

Hepatic miR-146a was increased in CHC patients compared to CHB patients. Recent data reported that miR-146a was promoting viral replication and deregulating metabolic pathways associated with liver disease^46^. The deregulation of miR-146a leading to the pathogenesis of liver disease may be specific of HCV infection. Interestingly, hepatic miR-122 was increased in CHC compared to CHB patients (Fig. 3). Whereas miR-122 stimulates HCV replication, miR-122 was suggested to inhibit the replication of HBV^21^. Therefore, in CHC hepatic miR-122 may be maintained to facilitate viral replication whereas in CHB miR-122 may be decreased to limit the inhibition of viral replication.

Serum miR-122 and miR-21 were weakly correlated with ALT and AST in CHB and CHC patients (Table 5). Moreover hepatic miR-122 was inversely correlated with AST and ALT. The current assumption regarding serum abundance of miRNAs is that miRNAs are released in the blood stream via an active and a passive release^47^. Thus, miR-21 and miR-122 may be passively released in the bloodstream following the lysis of hepatocytes. Interestingly, in CHB patients, while hepatic miR-122 was reduced compared to patients with CHC, serum miR-122 was 28 times higher than in CHC patients (Fig. 2N). These results illustrate the balance in between hepatic and serum miR-122. Since miR-122 is almost uniquely expressed in the liver, its presence in the serum is most probably due to a modification of its expression within hepatocytes. Serum miR-20a, miR-29a, miR-92a, miR-146a, miR-221, miR-222 and miR-224 were not correlated with AST or ALT suggesting that these miRNAs may be selectively released. However, besides miR-122 the miRNAs assessed in our study are expressed by multiple types of cells therefore their presence in the serum may be a consequence of a deregulation in other tissues. Interestingly, hepatic miR-20a and miR-92a were inversely correlated to the levels of ALT and AST (Table S9) whereas serum miR-20a and miR-92a were not correlated with AST and ALT (Table 5). These results support the hypothesis that the release of some miRNAs including miR-20a and miR-92a may be a consequence of the activation of different type of cells.

The expression of miR-21 in increased in pulmonary^48^ and renal fibrosis^49^. However, we found no up-regulation of hepatic miR-21 in CHB and CHC suggesting that miR-21 may not be involved in liver fibrosis.

When, we started this project, we made the decision to develop a selective approach rather than a global one for the following reasons: (i) because we support a physiopathology approach, (ii) because of the rarity of our serum and liver samples and finally (iii) because of cost issues. We do agree that this choice implies a limited number of miRNAs studied and that a global approach would have increased the number of hits. However, using this approach we developed 2 models to predict severe fibrosis and cirrhosis that remain both robust and relevant as compared to other tests such as APRI and FIB-4.

Our study supports the relevance to develop 2 models for the diagnosis of fibrosis specifically in patients with CHB and CHC. The model for patients with CHB (combining serum miR-122, serum miR-222, platelet count and ALP), and the model for patients with CHC (combining hepatic miR-122, hepatic miR-224, platelet count, ALT and albumin) were able to discriminate patients with F3-F4 from those with F1-F2. With better diagnostic performance than APRI and FIB-4. Interestingly, we report a differential profile of expression of miRNAs according to the stage of fibrosis and the type of viral infection. These findings may help to elucidate the molecular mechanisms of CHB and CHC-induced liver fibrosis.

## Methods

### Patients and Ethics statement

Among the 305 patients consecutively included, 103 and 178 had respectively CHB and CHC (Table S2). Patients were eligible if they had an established diagnosis of chronic viral hepatitis B or C (detectable HCV-RNA and HBV-DNA for more than 6 months) and had fibrosis assessment by liver biopsy. Patients were excluded if they had evidence of co-infection or any other cause of chronic liver disease. No patients were receiving treatment at the time of serum or liver biopsy. No patients have HCC. This study conformed to the ethical guidelines of the 1975 Declaration of Helsinki, All participants gave their informed written consent for the use of biological samples and clinical records. All experimental protocols were approved by the Comité de protection des personnes (CPP) and the Commission Nationale de l’Informatique et des Libertés (CNIL).

Among the CHB cohort, 87 serum and 83 liver samples were available for the analysis. Among the CHC cohort, 105 serum and 93 liver samples were available (Table S10). Interestingly, serum samples were coupled with liver samples for 69 and 20 patients of the CHB and CHC cohorts, respectively (Table S10). Previous non-responders to IFN, PEG-IFN plus ribavirin or boceprevir/telaprevir plus PEG-IFN plus ribavirin were included in the group of patients with CHC. 45 among 105 serums and 22 among 93 biopsies were from previous non-responders.

Patients with CHB were all naïve of treatment at the time of both the liver biopsy and the serum.

### Histopathological assessment of liver biopsies

Ultrasound guided percutaneous liver biopsies were performed. Liver biopsies were considered to be adequate if they were longer than 10 mm and/or had at least 12 portal tracts. Liver histology were all analyzed by an expert pathologist. The stage of fibrosis of liver biopsies was assessed according to the METAVIR score: F0. no fibrosis; F1, mild fibrosis (portal fibrosis without septa); F2, moderate fibrosis (portal fibrosis with rare septa); F3, advanced fibrosis (numerous septa without cirrhosis); F4, cirrhosis. Necroinflammation was assessed as follow: A0. no activity, A1, mild activity, A2, moderate activity, A3, severe activity^50^. Hepatic steatosis was graded according to the Brunt classification as: 0 (no hepatocytes affected by macrovesicular steatosis), 1 (0–33% hepatocytes affected), 2 (33–66% involved), and 3 (>66% hepatocytes affected)^51^.

### Non –invasive assessment of liver fibrosis

APRI and FIB-4 are scores derived from routine blood markers with are calculated as follow: FIB-4 = (age (year) × AST (IU/L))/(platelet count (10^9^/L) × ALT^1/2^ (IU/L))^52^, APRI = (AST (/ULN)/platelet count (10^9^/L)) × 100^11^.

A FIB-4 index less than 1.45 (Fib-4 < 1.45) predicts a Metavir score between F0 and F2 while a FIB-4 index equal to or above 3.25 (FIB-4 ≥ 3.25) predicts F3 and F4. In patients with a FIB-4 index ranging from 1.45 to 3.25 (1.45 < FIB-4 < 3.25), the stage of fibrosis cannot be determined and predicted accurately.

An APRI score less than or equal to 0.5 (APRI ≤ 0.5) predicts a Metavir score between F0 and F2. An APRI score equal to or above 1.5 (APRI ≥ 1.5) predicts a Metavir score of F3 or F4. In patients with an APRI score ranging from 0.5 to 1.5 (0.5 ≤ APRI ≤ 1.5), the stage of fibrosis cannot be determined and predicted accurately.

### Rationale of miRNAs selection

To select the miRNAs candidates, we conducted a selective research using pubmed. We use the following keywords: miRNAs, liver fibrosis, HCV and HBV either combined by two (miRNAs and liver fibrosis, miRNAs and HCV or miRNAS and HBV) or by three (miRNAs and liver fibrosis and HCV or miRNAs and liver fibrosis and HBV). The 13 miRNAs selected were either at least cited once in a major journal, or several times in minor journals. The 13 miRNAs selected were associated with: (1) the HCV and HBV life cycle and/or (2) the liver, cardiac and renal fibrosis and/or (3) the regulation of HSC activation and/or (4) HCC (Table S11).

MiR-20a and miR-92a are upregulated in serums of CHC patients compared to both healthy patients and those with fibrosis unrelated to HCV infection^53^. Serum and hepatic miR-21 is upregulated in patients with cirrhosis compared to healthy patients^54^. MiR-29 targets mRNAs of genes coding for extracellular matrix coding proteins and inhibits the proliferation of hepatic stellate cells (HSC). MiR-29 is inhibited by HCV and is down-regulated during fibrosis^55^. MiR-146a inhibits activation and proliferation of HSC and is down-regulated during fibrosis in mice models^56^. Increased expression of miR-221 and miR-222 indicates the activation of HSC and the progression of liver fibrosis^27^. Increased expression of miR-224 was associated with the development of HCC^43^,^44^ (Table S11).

Chronic hepatitis B and C are main causes of hepatocellular carcinoma. Deregulation of several miRNAs has been associated with hepatocellular carcinoma^57^,^58^,^59^. Therefore, we investigated the potential of miRNAs related to hepatocellular carcinoma to be associated with the different stages of fibrosis. We assessed the expression of miR-26a/-26b, miR-199a/b-3p and miR-223 in serum and liver samples of patients with CHB or CHC (Supplementary Figures 5 and 6 and Tables S12 and S13). None of these miRNAs was significantly deregulated in patients with F3-F4 compared to those with F0-F1-F2 (Supplementary Figures 5 and 6).

### Total RNAs extraction from biopsies and serums

#### From liver tissues

Frozen liver biopsy specimens were crushed and dissolved in 1ml of iced RNAble (Eurobio). Chloroform was added; the upper phase was collected after centrifugation and added to the same volume of isopropanol. RNA was precipitated, and washed in 80% Et-OH. The pellet was dissolved in RNase and DNase free H_2_O^60^. Optical density of total RNA was measured at 260 nm.

#### From serums

Total RNAs were extracted from 400 μl of serum (MiRvana, Life science). Since there is no reliable serum miRNA stably expressed in the serum, 5.6 × 10^8^ copies of *Caenorhabdditis elegans* miR-39 (cel-miR-39, Qiagen) was added to each serum sample before the extraction^20^,^60^.

### MiRNAs quantification

The miRNAs content in both liver and serum samples was analysed by Taqman RT-qPCR. Quantitative values are obtained from the crossing point (Cp), number at which the increase in the signal associated with exponential growth of PCR products begins to be detected using Light Cycler^®^ 480 System Software (Roche Diagnostics), according to the manufacturer’s manuals. Briefly, 1 ng of the reverse transcription product was used to assess the expression of each miARNs. The expression of hepatic microRNAs was normalized to the amount of a control small RNA, SNORD44^20^,^60^ whereas the amount of serum microRNAs was normalized to the amount of the spiked-in *cel*-miR-39.

All the results were expressed as the N-fold differences in target gene expression relative to SNORD44 and cel-miR-39, and termed Ntarget. Ntarget was determined as Ntarget = 2ΔCpsample. The ΔCp value was determined by subtracting the average Cp value of the target miRNA from the average Cp value of SNORD44 (liver biopsy samples) and *cel-*miR-39 (serum samples).

### Statistical analysis

Data are presented as medians and interquartile range for continuous variables and frequencies with percentages for qualitative variables. Marginal association between single variables and F3-F4 fibrosis was assessed by a Wilcoxon rank-sum test for quantitative variables and Fisher’s exact test. Multiple logistic regressions were used to determine a set of variables independently associated with F3-F4 fibrosis. Variables associated with intervention at a 0.05 level were considered in the multiple models. The model selections were performed using a backward stepwise variable algorithm and Akaike Criteria. Odds Ratio (OR) are given, with their 95% Confidence intervals (95% CI). Performance of models and markers was assessed using Area under the Curve (AUC) and its 95% CI. Cut-off values for the prediction of patients with F3-F4 fibrosis was determined by maximizing the Youden index. Sensitivity, Specificity were directly estimated with their 95% CI. Predictive positive value (PPV) and Negative predictive value were estimated using a prevalence of F3-F4 fibrosis in the population of 20%. All tests were 2 sided, and a value P < 0.05 was considered statistically significant. Statistical analyses were carried out with R (version 3.1.0).

## Additional Information

**How to cite this article**: Appourchaux, K. *et al*. MicroRNA-based diagnostic tools for advanced fibrosis and cirrhosis in patients with chronic hepatitis B and C. *Sci. Rep.* **6**, 34935; doi: 10.1038/srep34935 (2016).

## Supplementary Material

Supplementary Information

## Figures and Tables

**Table 1:** Characteristics of the patients according to the etiology of the liver disease (CHB and CHC).

N, patients	Chronic hepatitis B					Chronic Hepatitis C				
	N total = 58	F0/F1/F2	N total = 44	F3/F4	p value	N total = 84	F0/F1/F2	N total = 94	F3/F4	p value
Gender: male/female, n (%)	58	50 (86.2)/8 (13.8)	44	37 (84.1)/7 (15.9)	0.78	84	41 (48.8)/43 (51.2)	94	64 (68.1)/30 (31.9)	0.01
Age, years (median, IQ)	58	42 [30.25; 52.5]	43	42 [33.5; 47]	0.83	84	49 [43; 55]	92	49 [44; 54]	0.94
BMI, kg.m^−2^ (median, IQ)	50	25.1 [23.1; 27.5]	41	25.9 [23.2; 28.3]	0.43	70	24.6 [22.2; 27.8]	64	25.8 [24.2; 28.8]	0.045
ALT, IU/L (median, IQ)	58	66 [49; 120.8]	44	78.5 [54.8; 161]	0.50	82	79.5 [57.2; 101.5]	92	119 [65.5; 154.5]	0.0002
AST, IU/L (median, IQ)	58	40.5 [34.3; 62.8]	44	59.5 [36; 131]	0.047	82	47 [36.5; 62.8]	91	77 [52; 112]	<0.0001
ALP, IU/L (median, IQ)	57	43 [27; 63]	40	94 [44.5; 181.5]	0.0002	82	65 [52; 78.8]	88	80 [60.8; 107]	<0.0001
GGT, IU/L (median, IQ)	55	15 [11; 17]	43	18 [14.5; 24.5]	0.001	82	44.5 [26; 78.5]	90	96 [58.25; 156]	<0.0001
Platelets, x10^3^/mm^3^(median, IQ)	58	196.5 [162; 234.8]	43	156 [135; 192]	0.0003	78	230 [188; 263]	83	172 [120.5; 209]	<0.0001
Cholesterol, mmol/L (median, IQ)		NA		NA		68	4.75 [4.3; 5.5]	60	4.2 [3.6; 4.7]	<0.0001
Triglycerides, mmol/L (median, IQ)	50	0.9 [0.6; 1.1]	32	1.0 [0.7; 1.7]	0.035	67	0.94 [0.7; 1.1]	59	0.92 [0.7; 1.4]	0.44
Glycemia, mmol/L (median, IQ)	51	4.8 [4.3; 5.1]	35	5.1 [4.5; 5.9]	0.027	71	4.9 [4.5; 5.4]	71	5.4 [4.8; 6.1]	0.0003
Total bilirubin, μmol/L (median, IQ)	22	10.1 [6.3; 13.8]	6	16,9 [13.6; 39.5]	0.031	79	12 [9; 15]	90	14 [10; 18]	0.01
Albumin, g/L (median, IQ)	57	47.2 [44; 49.4]	41	44.4 [41.4; 47.3]	0.002	69	45.4 [44; 48]	70	45.1 [43; 47.8]	0.26
Viral Loads, logUI/mL (median, IQ)	58	6.2 [4.3; 7.3]	42	5.9 [4.1; 7.2]	0.27	52	5.6 [5.2; 6.1]	59	5.9 [5.3; 6.3]	0.10
Necroinflammatory activity, n (%)	58		44		0.016	83		89		0.001
None (A0)		4 (6.9)		1 (2.3)			10 (11.9)		5 (5.3)	
Mild (A1)		33 (56.9)		18 (40.9)			58 (69.0)		44 (46.8)	
Moderate (A2)		20 (34.5)		20 (45.4)			14 (16.7)		35 (37.2)	
Severe (A3)		1 (1.7)		5 (11.4)			1 (1.2)		5 (5.3)	
NA		0		0			1 (1.2)		5 (5.3)	
Steatosis Grades, n (%)	58		44		0.26	82		84		0.02
0		35 (60.3)		22 (50)			37 (44.05)		23 (24.5)	
1		15 (25.9)		12 (27.3)			27 (32.14)		24 (25.5)	
2		5 (8.6)		8 (18.1)			15 (17.86)		28 (29.8)	
3		0 (0)		1 (2.3)			3 (3.57)		9 (9.6)	
NA		3 (5.2)		1 (2.3)			2 (2.38)		10 (10.6)	
										
HCV genotypes, n (%)		—		—		78		88		0.007
1		—		—			40 (47.6)		48 (51.1)	
2		—		—			11 (13.1)		2 (2.1)	
3		—		—			11 (13.1)		17 (18.1)	
4		—		—			11 (13.1)		21 (22.3)	
5		—		—			3 (3.6)		0 (0)	
6		—		—			1 (1.2)		0 (0)	
NA		—		—			7 (8.3)		6 (6.4)	

AST, aspartate aminotransferase; ALT, alanine aminotransferase; ALP, alkaline phosphate; BMI, Body mass index; GGT, Gamma-glutamyltranspeptidase, IQ: Interquartile range, NA: Not available. Clinical parameters are expressed as median and interquartile range (IQ), unless indicated differentially. Differences between chronic hepatitis B and C patients were evaluated with the Fisher’s exact (qualitative variables) and the Wilcoxon rank-sum tests (continuous variables).

**Table 2:** Multivariate analysis of clinical data and serum microRNA and their association with fibrosis in patients with CHB and CHC.

		OR	95% CI	p value
CHB patients	Serum miR-122	0.19	0.03–1.17	0.07
	Serum miR-222 (x100)	1.12	1.01–1.24	0.03
	Platelets	0.99	0.98–1.00	0.09
	ALP (/10)	1.21	1.04–1.41	0.01
CHC patients	Hepatic miR-122	0.80	0.67–0.95	0.01
	Hepatic miR-224 (x100)	1.56	1.05–2.31	0.03
	Platelets	0.97	0.95–0.99	0.004
	ALT (/10)	1.09	0.96–1.24	0.17
	Albumin	1.41	0.94–2.11	0.10

**Table 3:** Diagnostic performance of the miRNAs-based models to diagnose F3–F4 fibrosis in patients with CHB and CHC.

CHB model	AUC	95% CI	p value
Serum miR-122	0.85	0.77–0.95	
Serum miR-222			
Platelet count			
ALP			
APRI	0.70	0.58–0.82	0.007
FIB-4	0.81	0.71–0.90	0.39
Hepatic miR-122	0.93	0.87–0.99	
Hepatic miR-224			
Platelet count			
ALT			
Albumin			
APRI	0.86	0.75–0.96	0.11
FIB-4	0.79	0.67–0.90	0.009

**Table 4:** Analytical performance of APRI, FIB-4 and our miRNA-based models to diagnose fibrosis in CHB and CHC patients.

Etiology	Score	Cut-off value	Sensitivity (95% CI)	Specificity (95% CI)	PPV	NPV
CHB patients	FIB-4	1.45	0.67 (0.51–0.79)	0.72 (0.60–0.83)	0.38	0.90
		3.25	0.26 (0.14–0.4)	0.97 (0.91–1)	0.65	0.84
	APRI	0.5	0.84 (0.72–0.93)	0.43 (0.31–0.55)	0.27	0.91
		1.5	0.35 (0.21–0.49)	0.9 (0.81–0.97)	0.46	0.85
	Our miRNA-based signature	0.57	0.79 (0.71–0.88)	0.95 (0.92–0.97)	0.79	0.95
CHC patients	FIB-4	1.45	0.76 (0.66–0.85)	0.67 (0.56–0.77)	0.36	0.92
		3.25	0.27 (0.18–0.37)	0.99 (0.96–1)	0.84	0.84
	APRI	0.5	0.91 (0.85–0.98)	0.4 (0.29–0.51)	0.27	0.95
		1.5	0.38 (0.28–0.48)	0.95 (0.90–0.99)	0.65	0.86
	Our miRNA-based signature	0.32	0.89 (0.86–0.92)	0.84 (0.80–0.88)	0.58	0.97

A cut-off value for the prediction of patients with F3–F4 fibrosis was determined by maximizing the Youden index. The optimal cut-off values were assessed by the sensitivity, specificity, positive (PPV) and negative predictive values (NPV). PPV and NPV were calculated as follow: (Prevalence of patients with F3-F4 among the total population of patients with viral hepatitis is: 20%).

PPV = [sensitivity × prevalence]/[sensitivity × prevalence + (1−specificity) × (1−prevalence)].

NPV = [specificity × (1 − prevalence)]/[(1 − sensitivity) × prevalence + specificity × (1 − prevalence)].

**Table 5:** Correlations between the expression of serum microRNAs and ALT and AST in CHB and CHC patients.

	Chronic hepatitis B				Chronic hepatitis C			
	ALT		AST		ALT		AST	
	R^2^	p-value	R^2^	p-value	R^2^	p-value	R^2^	p-value
miR-20a	0.17	0.11	0.13	0.24	−0.08	0.45	−0.04	0.68
miR-21	**0.23**	**0.04**	**0.23**	**0.04**	0.07	0.51	0.15	0.14
miR-27a	0.08	0.56	0.09	0.51				
miR-27b	0.24	0.05	0.19	0.14				
miR-29a	0.16	0.15	0.11	0.31	0.10	0.39	0.13	0.27
miR-29c	0.14	0.23	0.11	0.33				
miR-92a	0.08	0.49	−0.02	0.86	−0.05	0.66	−0.05	0.61
miR-122	**0.28**	**0.01**	0.18	0.10	**0.28**	**0.01**	0.31	0.009
miR-146a	0.01	0.92	0.07	0.55	0.03	0.80	0.00	0.99
miR-155	−0.01	0.95	0.07	0.57				
miR-221	0.18	0.15	0.18	0.14	0.00	0.98	0.03	0.82
miR-222	0.16	0.14	0.15	0.16	0.02	0.87	0.03	0.75
miR-224	0.05	0.75	−0.03	0.84	0.13	0.44	0.10	0.55
