# Supplementary material for: MicroRNA-based diagnostic tools for advanced fibrosis and cirrhosis in patients with chronic hepatitis B and C
Source: Sci Rep. 2016 Oct 12;6:34935. doi: 10.1038/srep34935 (PMC5059729; doi:10.1038/srep34935)

MicroRNA-based diagnostic tools for advanced fibrosis and cirrhosis

In patients with chronic hepatitis B and C

Kevin Appourchaux, Safi Dokmak, Matthieu Resche-Rigon, Xavier Treton, Martine Lapalus, Charles-Henry Gattolliat, Emmanuelle Porchet, Michelle Martinot-Peignoux, Nathalie Boyer, Michel Vidaud, Pierre Bedossa, Patrick Marcellin, Ivan Bièche, Emilie Estrabaud and Tarik Asselah.

Supplementary Tables:

Table S1: Characteristics of chronic hepatitis B and C patients with fibrosis F0-F1-F2 and F3-F4.

	F0-F1-F2	F3-F4	p values
n, patients	142	138	
Gender: male / female, n (%)	91 (64.1) / 51 (35.9)	101 (31.9) / 37 (68.1)	0.12
Age, years (median, IQ)	47 [38 ; 54]	47 [41.5 ; 53]	0.60
BMI, kg.m^{-2} (median, IQ)	24.9 [22.5 ; 27.6]	25.9 [23.9 ; 28.7]	0.04
ALT IU/L (median, IQ)	77 [50 ; 110.2]	97 [61 ; 155.5]	0.001
AST IU/L (median, IQ)	46 [35 ; 63]	72 [49 ; 113.5]	<0.0001
ALP IU/L (median, IQ)	60 [43 ; 76]	80 [56 ; 113.5]	<0.0001
GGT IU/L (median, IQ)	24 [15 ; 50]	59 [23 ; 120]	<0.0001
Platelets, x10^3/mm^3 (median, IQ)	216 [177.8 ; 251.8]	169 [131.8 ; 202]	<0.0001
Cholesterol, mmol/L (median, IQ)	4.76 [4.3 ; 5.6]	4.2 [3.628 ; 4.7]	<0.0001
Triglycerides, mmol/L (median, IQ)	0.88 [0.6 ; 1.1]	0.94 [0.71 ; 1.6]	0.045
Glycemia, mmol/L (median, IQ)	4.8 [4.4 ; 5.2]	5.3 [4.7 ; 6.1]	<0.0001
Total Bilirubine (median, IQ)	11 [8 ; 15]	14 [10 ; 18]	0.001
Albumine g/L (median, IQ)	46.45 [44 ; 49]	45 [42.35 ; 47.4]	0.004

AST, aspartate aminotransferase; ALT, alanine aminotransferase; ALP, alkaline phosphate; BMI, Body mass index; GGT, Gamma-glutamyl transpeptidase, IQ: Interquartile range, NA: Not available.

Clinical parameters are expressed as median and interquartile range (IQ), unless indicated differentially. Differences between chronic hepatitis B and C patients were evaluated with the Fisher exact (qualitative variables) and the Wilcoxon rank-sum test (continuous variables).

Table S2: Characteristics of patients with chronic hepatitis B and C

	Chronic hepatitis B	Chronic hepatitis C	p values
n, patients	103	178	
Gender: male / female, n (%)	88 (85.4) / 15 (14.6)	105 (58.9) / 73 (41.1)	<0.0001
Age, years (median, IQ)	42 [31.2 ; 50.5]	49 [43 ; 54]	<0.0001
BMI, $kg.m^{-2}$ (median, IQ)	25.1 [23.1 ; 27.8]	25.2 [23.1 ; 28.5]	0.85
ALT, IU/L (median, IQ)	71 [49 ; 126.5]	88 [61 ; 133]	0.09
AST, IU/L (median, IQ)	49 [35 ; 84.5]	59 [43 ; 88]	0.03
ALP, IU/L (median, IQ)	50 [33 ; 94]	71.5 [56 ; 91.8]	0.0003
GGT, IU/L (median, IQ)	16 [12 ; 19.8]	63.5 [39 ; 132.2]	<0.0001
Platelets, $x10^3/mm^3$ (median, IQ)	180 [148 ; 223]	195 [159 ; 245]	0.04
Triglycerides, mmol/L (median, IQ)	0.88 [0.62 ; 1.4]	0.93 [0.7 ; 1.2]	0.45
Glycemia, mmol/L (median, IQ)	4.8 [4.3 ; 5.3]	5 [4.6 ; 5.5]	0.01
Total bilirubin, µmol/L (median, IQ)	11.2 [6.8 ; 16.8]	13 [9 ; 16]	0.25
Albumin, g/L	46.4 [42.5 ; 48.6]	45.4 [43.4 ; 48]	0.58
Necroinflammatory activity, n (%)			0.14
None (A0)	5 (4.8)	15 (8.43)	
Mild (A1)	51 (49.5)	102 (57.30)	
Moderate (A2)	40 (38.8)	49 (27.53)	
Severe (A3)	6 (5.83)	6 (3.37)	
NA	1 (0.98)	6 (3.37)	
Steatosis grade, n (%)			0.001
0	57 (55.35)	60 (33.71)	
1	27 (26.21)	51 (28.65)	
2	13 (12.62)	43 (24.16)	
3	1 (0.97)	12 (6.74)	
NA	4 (4.85)	12 (6.74)	
Fibrosis stage (Metavir), n (%)			0.23
F0	2 (1.94)	0 (0)	
F1	28 (27.18)	45 (25.28)	
F2	28 (27.18)	39 (21.91)	
F3	23 (22.33)	53 (29.78)	
F4	21 (20.39)	41 (23.03)	
NA	1 (0.98)	0 (0)	

AST, aspartate aminotransferase; ALT, alanine aminotransferase; ALP, alkaline phosphate; BMI, Body mass index; GGT, Gamma-glutamyl transpeptidase, IQ: Interquartile range, NA: Not available.

Clinical parameters are expressed as median and interquartile range (IQ), unless indicated differentially. Differences between chronic hepatitis B and C patients were evaluated with the Fisher exact (qualitative variables) and the Wilcoxon rank-sum test (continuous variables).

Table S3: Characteristics of patients with CHB and CHC who have been treated.

	CHB (n=7)		CHC (n=9)	
	Before	After	Before	After
Age, years (median, IQ)	49.5 (37.25-55.0)	50.5 (38.25-56.0)		
Time between the initiation of treatment and the second serum, months (median, IQ)		12.5 (11.5-14)		12.0 (9-13)
ALT, IU/L (median, IQ)	44.5 (37-55.75)	28 (24-47.5)	83 (60-130)	30 (25-32)
AST, IU/L (median, IQ)	37 (34.25-62.25)	24 (23-38)	58 (50-111)	25 (24-32)
ALP, IU/L (median, IQ)	71 (66-82)	66 (57-75)	60 (53.25-71.75)	91 (82.5-114)
GGT, IU/L (median, IQ)	49.5 (46-54.5)	33 (28-33)	63 (39-132)	21 (19.75-49.50)
Fibrosis at the first serum (Metavir)	F2: 3; F3: 3, NA:1		F3:7; F4:2	
Necro-inflammation activity at the first serum (Metavir)	A1: 4; A2: 2; NA: 1		A1: 6; A2:3	
Viral Load, logUI/mL (median, IQ)	3.94 (3.21-4.24)	Undetectable	5.84 (5.61-6.06)	Undetectable

AST, aspartate aminotransferase; ALT, alanine aminotransferase; ALP, alkaline phosphate; GGT, Gamma-glutamyl transpeptidase, IQ: Interquartile range, NA: Not available.

Clinical parameters are expressed as median and interquartile range (IQ), unless indicated differentially.

Table S4: Characteristics of the patients with chronic hepatitis B and C for whom serum samples are available.

	**Chronic hepatitis B**					**Chronic Hepatitis C**				
		**F0/F1/F2**		**F3/F4**	**p value**		**F0/F1/F2**		**F3/F4**	**p value**
N, patients	N total=49		N total=38			N total=42		N total=63		
Gender: male / female, n (%)	49	42 (85.71%)/ 7(14.29%)	38	32 (84.21%)/ 6(15.79%)	1.00	42	13 (45.24)/ 23 (54.76%)	63	46 (73.02%)/17 (26.98%)	0.007
Age, years (median, IQ)	49	41 [30 ;49]	38	40.5 [33.25 ;47]	0.71	42	47.5 [39.5 ;53]	61	50 [46 ;54]	0.15
BMI, kg.m^{-2} (median, IQ)	45	1.75 [1.69 ;1.81]	36	1.72 [1.657 ;1.76]	0.13	33	23.44 [22.05 ;27.78]	35	26.61 [24.33 ;28.88]	0.008
ALT, IU/L (median, IQ)	49	73 [49 ;130]	38	78.5 [48.75 ;191.8]	0.90	40	79 [58.5 ;96.75]	61	119 [72 ;147]	0.005
AST, IU/L (median, IQ)	49	42 [34 ;70]	38	59.5 [36 ;128.8]	0.14	40	50 [41 ;66]	61	78 [53 ;113]	<0.0001
ALP, IU/L (median, IQ)	49	43 [25 ;61]	35	94 [39.5 ;164]	0.0003	40	67 [50 ;79.25]	59	80 [60.5 ;104.5]	0.005
GGT, IU/L (median, IQ)	49	15 [11 ;17]	37	17 [16 ;25]	0.002	40	51 [23.5 ;135]	60	107 [62.75 ;166.2]	0.001
Platelets, x10^3/mm^3(median, IQ)	49	206 [175 ;234]	37	156 [136 ;193]	0.0003	36	224 [189.5 ;262.2]	53	172 [107 ;208]	<0.0001
Cholesterol, mmol/L (median, IQ)						28	4.675 [4.22 ;5.438]	36	4.295 [3.505 ;4.712]	0.017
Triglycerides, mmol/L (median, IQ)	43	0.85 [0.56 ;1.12]	28	1.01 [0.6675 ;1.612]		27	1.01 [0.74 ;1.375]	35	1.07 [0.745 ;1.625]	0.63
Glycemia, mmol/L (median, IQ)	45	4.8 [4.3 ;5.1]	30	5.05 [4.5 ;5.5]	0.049	31	4.8 [4.45 ;5.15]	45	5.5 [4.96 ;6.6]	0.0001
Total bilirubin, µmol/L (median, IQ)	18	10.45 [6.1 ;13.75]	5	16.7 [12.5 ;17.2]	0.093	37	10 [8 ;14]	60	14.5 [9.75 ;18.25]	0.0009
Albumin, g/L (median, IQ)	48	47.05 [43.65 ;49.32]	35	44.1 [41.5 ;47.2]	0.007	27	45.4 [43.8 ;47.7]	42	44.5 [42.85 ;48]	0.42
Viral Loads, logUI/mL	49	6.261 [4.417	37	5.977 [4.348	0.35	25	5.892 [5.238	36	5.96 [5.389	0.37

(median, IQ)		;7.516]		;7.253]			;6.273]		;6.419]	
Necroinflammatory activity, n (%)	49		38		0.12	41		58		<0.0001
None (A0)		4 (8.16 %)		1 (2.63 %)			6 (14.63%)		0	
Mild (A1)		27 (55.1 %)		16 (42.11 %)			31 (75.61%)		28 (48.28 %)	
Moderate (A2)		17 (34.69 %)		16 (42.11 %)			4 (9.76%)		26 (44.83 %)	
Severe (A3)		1 (2.04 %)		5 1(3.16 %)			0		4 (6.9 %)	
NA		0		0			1		5	
Steatosis Grades, n (%)	49		38		0.55	40		54		0.32
0		29 (59.18 %)		19 (50 %)			15 (37.5 %)		13 (24.07 %)	
1		12 (24.49 %)		10 (26.32 %)			14 (35 %)		17 (31.48 %)	
2		5 (10.2 %)		7 (18.42 %)			9 (22.5 %)		17 (31.48 %)	
3		0		1 (2.63 %)			2 (5 %)		7 (12.96 %)	
NA		3 (6.12 %)		1 (2.63 %)			2		9	
HCV genotypes, n (%)		-		-		37		57		0.13
1		-		-			18 (48.65 %)		31 (54.39 %)	
2		-		-			5 (13.51 %)		1 (1.75 %)	
3		-		-			7 (18.92 %)		12 (21.05 %)	
4		-		-			6 (16.22 %)		13 (22.81 %)	
5		-		-			1 (2.7 %)		0	
6		-		-			0		0	
NA		-		-			5		6	

AST, aspartate aminotransferase; ALT, alanine aminotransferase; ALP, alkaline phosphate; BMI, Body mass index; GGT, Gamma-glutamyl transpeptidase, IQ: Interquartile range, NA: Not available.

Clinical parameters are expressed as median and interquartile range (IQ), unless indicated differentially. Differences between patients with F0-F1-F2 and those with F3-F4 were evaluated with the Fisher exact (qualitative variables) and the Wilcoxon rank-sum test (continuous variables).

Table S5: Patients stratified according to their fibrosis stage and FIB-4 score

	FIB-4								
	CHB			CHC			CHB+CHC		
	F0-F1-F2	F3-F4	p-values	F0-F1-F2	F3-F4	p-values	F0-F1-F2	F3-F4	p-values
FIB-4 < 1.45	42	14	<.0001	52	19	<.0001	94	33	<.0001
1.45<FIB-4<3.25	14	18		25	39		39	57	
FIB-4 ≥ 3.25	2	11		1	21		3	32	
NA	1	1		6	15		7	16	

NA: Not available.

Table S6: Patients stratified according to their fibrosis stage and APRI score

	APRI								
	CHB			CHC			CHB+CHC		
	F0-F1-F2	F3-F4	p-values	F0-F1-F2	F3-F4	p-values	F0-F1-F2	F3-F4	p-values
APRI ≤ 0.5	25	7	0.002	32	7	<0.001	57	14	<0.001
0.5<APRI<1.5	27	21		42	43		69	64	
APRI ≥ 1.5	6	15		4	30		10	45	
NA	1	1		6	14		7	15	

NA: Not available.

Table S7: Characteristics of the patients with chronic hepatitis B and C for whom liver biopsies samples are available.

	Chronic hepatitis B					Chronic Hepatitis C				
		**F0/F1/F2**		**F3/F4**	**p value**		**F0/F1/F2**		**F3/F4**	**p value**
N, patients	N total= 48		N total=35			N total=53		N total=40		
Gender: male / female, n (%)	48	40 (83.33%)/8(16.67%)	35	28 (80.0%)/7 (20.0%)	0.78	53	28 (52.83%)/ 25 (47.17%)	40	23 (57.5%)/17 (42.5%)	0.68
Age, years (median, IQ)	48	42.5 [30 ; 53.25]	34	40.5 [33.25 ;45.75]	0.58	53	51 [45 ;56]	40	48.5 [43.75 ;52.25]	0.26
BMI, kg.m^{-2} (median, IQ)	41	25.11 [23.66 ;27.47]	32	26.12 [23.45 ;29.59]	0.40	47	25.25 [23.16 ;27.81]	37	25.25 [23.97 ;28.94]	0.44
ALT, IU/L (median, IQ)	48	63.5 [47.75 ;119]	35	74 [56 ;140]	0.33	53	77 [57 ;99]	39	87 [63 ;171]	0.055
AST, IU/L (median, IQ)	48	39 [33.75 ;59]	35	61 [36.5 ;101]	0.031	53	46 [36 ;61]	39	71 [49.5 ;91]	0.0005
ALP, IU/L (median, IQ)	47	37 [26 ;62]	32	100 [52.5 ;209]	<0.0001	53	64 [52 ;77]	38	80 [62.5 ;107]	0.002
GGT, IU/L (median, IQ)	45	15 [11 ;18]	34	17 [16 ;24.75]	0.003	53	41 [28 ;63]	39	87 [54 ;160]	<0.0001
Platelets, x10^3/mm^3(median, IQ)	48	188.5 [159.8 ;234.2]	34	155.5 [135.2 ;179.8]	0.001	53	230 [187.5 ;263]	38	179.5 [140.5 ;210]	<0.0001
Cholesterol, mmol/L (median, IQ)						50	4.845 [4.295 ;5.512]	30	3.965 [3.562 ;4.615]	<0.0001
Triglycerides, mmol/L (median, IQ)	42	0.83 [0.5425 ;1.12]	27	1.07 [0.69 ;1.795]	0.029	50	0.925 [0.7075 ;1.062]	30	0.855 [0.7025 ;1.09]	<0.0001
Glycemia, mmol/L (median, IQ)	41	4.8 [4.3 ;5.1]	29	5.1 [4.5 ;5.6]	0.046	50	4.9 [4.425 ;5.3]	32	5.1 [4.675 ;5.625]	0.11
Total bilirubin, µmol/L (median, IQ)	18	8.75 [6.275 ;13.03]	5	17.2 [12.5 ;46.9]	0.040	52	13 [10 ;15.25]	39	12 [10 ;16.5]	0.83
Albumin, g/L (median, IQ)	47	47.6 [45.9 ;49.45]	32	44.25 [41.5	0.0006	52	45.3 [44	36	46 [43.85	0.82

				;47.15]			;47.47]		;48]	
Viral Loads, logUI/mL (median, IQ)	48	5.879 [4.203 ;7.253]	33	5.82 [3.689 ;6.903]	0.36	34	5.524 [5.222 ;5.972]	30	5.98 [5.13 ;6.207]	0.20
Necroinflammatory activity, n (%)	48		35		0.046	53			40	0.22
None (A0)		3 (6.25%)		1 (2.86%)			6 (11.32%)		5 (12.5%)	
Mild (A1)		27 (56.25%)		16 (45.71%)			34 (64.15%)		19 (47.5%	
Moderate (A2)		18 (37.05%)		13 (37.14%)			12 (22.64%)		14 (35%)	
Severe (A3)		0		5 (14.29%)			1 (1.89%)		2 (5.26%)	
NA		0		0			0		2	
Steatosis Grades, n (%)	48		35		0.20	53		38		0.058
0		31 (64.58%)		18 (51.43%)			26 (49.06%)		14 (36.84%)	
1		11 (22.92%)		9 (25.71%)			17 (32.08%)		8 (21.05%)	
2		4 (8.33%)		6 (17.14%)			9 (16.98%)		14 (36.84%)	
3		0		1 (2.86%)			1 (1.89%)		2 (5.26%)	
NA		2 (4.17%)		1 (2.86%)			0		2	
HCV genotypes, n (%)		-		-		52		35		0.14
1		-		-			28 (56 %)		23 (57.5 %)	
2		-		-			9 (18 %)		2 (5 %)	
3		-		-			4 (8 %)		5 (12.5 %)	
4		-		-			6 (12 %)		10 (25 %)	
5		-		-			2 (4 %)		0	
6		-		-			6 (1 2 %)		0	
NA		-		-			3		0	

AST, aspartate aminotransferase; ALT, alanine aminotransferase; ALP, alkaline phosphate; BMI, Body mass index; GGT, Gamma-glutamyl transpeptidase, IQ: Interquartile range, NA: Not available.

Clinical parameters are expressed as median and interquartile range (IQ), unless indicated differentially. Differences between patients with F0-F1-F2 and those with F3-F4 were evaluated with the Fisher exact (qualitative variables) and the Wilcoxon rank-sum test (continuous variables).

Table S8: Correlations between the level of expression of hepatic and serum microRNA

	R^2	p value
miR-20a	**0.31**	**0.01**
miR-21	0.14	0.30
miR-27a	0.07	0.63
miR-27b	-0.07	0.65
miR-29a	-0.03	0.82
miR-29c	0.01	0.96
miR-92a	0.07	0.57
miR-122	0.00	1.00
miR-146a	**0.36**	**0.009**
miR-155	0.10	0.46
miR-221	0.21	0.20
miR-222	0.18	0.13
miR-224	0.23	0.15

Table S9: Correlations between the hepatic expression of miR-20a, miR-92a and miR-122 and ALT and AST levels

Liver biopsies	Chronic hepatitis C			
	ALT		AST	
	R^2	p-value	R^2	p-value
miR-20a	-0.31	0.009	-0.36	0.009
miR-92a	-0.25	0.02	-0.27	0.01
miR-122	-0.25	0.01	-0.35	0.009

Table S10: Number of serums and biopsies studied in each etiology (CHB or CHC)

	CHB	CHC
Serums	87	105
Liver biopsies	83	93
Coupled serums/liver biopsies	69	20

Table S11: Description of the selected miRNAs.

miRNA	Targets	Expression induced by	Regulation of Signaling pathways	Organs	Roles in the liver	Other Description	References
miR-20a			PTEN/PI3K/Akt	Liver, lung, kidney	HCC	Circulating miR-20a is correlated with HCV-mediated liver disease<br>Biomarker of HCC	[1-3]
miR-21	Smad7, PTEN, PPARα,<br>PDCD4,<br>BTG2	NF-KB pathway, HBx	TGF- β, ERK/MAPK,<br>HBP1-p53-Srebp1c pathway.	lung, heart, kidney	Lipid oxydation, Mitochondria functions, Inhibition of chrolesterol synthesis, HSC proliferation, ECM production, inflammation, Pathogenesis of NAFLD	Biomarker (serum miR-21) of necroinflammation<br>Quiescent hepatocytes express high levels of miR-21<br>Potential link between fatty liver disease and HCC<br>Increased expression in pulmonary and renal fibrosis	[4]<br>[5-16]
miR-27a, b	FOXO1		Wnt/ β-catenin	Liver	HSC proliferation, steatosis, lipid metabolism, Inhibition of chrolesterol synthesis		[8, 17-19]

miR-29a, c	Collagens (I, III, IV, V, VII, VIII), FBN1, ELN1, MMP2, PDGF, IGF-1, MCT1, INSIG1, CAV2	HBx	TGF-Beta, PI3K/Akt, Wnt/ β-catenin, NF-KB	Heart, lung, kidney, liver	Inhibition of ECM production, inhibition of neogluconeogenesis		[20-24]
miR-92a	PTEN		TGF-Beta	Liver, heart, lung	HCC		[1, 2]
miR-122	Cycline G1, KLF6, CAT1, ADAM17 P4HA1n, Bcl-w		IFN signaling pathway, Akt, p53	Liver	Antifibrotic activity, HCC	Biomarker of necroinflammation (Correlations between serum miR-21 and miR-122 levels and ALT)<br>Serum miR-122 is increased in CHC patient compared to healthy controls	[4, 25-29]
miR-146a-5p	SMAD4, Wnt1, Wnt5a	NF-KB pathway, HBx	Notch and Wnt pathways	Liver, Kidney	Inhibition of HSC, inflammation,		[30-33]
miR-155	PPRE, PPARα		TNF-alpha	Lung, Liver	Inflammation, Profibrotic		[34]
miR-221	DDIT4, TIMP3	β-catenine<br>(HBx)	NF-KB, mTOR	Liver, Kidney	HCC, lipid metabolism, proliferation of HSC, inhibition of HSC apoptosis,		[35]<br>[36-38]

					liver cancer		
miR-222	HDAC-4	HBx	NF-KB, mTOR	Liver	Activation and proliferation of HSC, inhibition of MMP-13 production, HCC		[15, 37, 39, 40]
miR-224	SMAD4, p21, p15, CCNE1		PI3K/Akt, Wnt/ β-catenin	Liver, Kidney	HCC, steatosis		[15, 41-43]

References:

1. **Kodama, T. et al. Increases in p53 expression induce CTGF synthesis by mouse and human hepatocytes and result in liver fibrosis in mice. *The Journal of clinical investigation* 121, 3343-3356 (2011).**
2. **Shrivastava, S. et al. Up-regulation of circulating miR-20a is correlated with hepatitis C virus-mediated liver disease progression. *Hepatology* 58, 863-871 (2013).**
3. **Wen, Y. et al. Plasma miRNAs as early biomarkers for detecting hepatocellular carcinoma. *International journal of cancer* 137, 1679-1690 (2015).**
4. **Migita, K. et al. Circulating microRNA Profiles in Patients with Type-1 Autoimmune Hepatitis. *PloS one* 10, e0136908 (2015).**
5. **McClelland, A.D. et al. miR-21 promotes renal fibrosis in diabetic nephropathy by targeting PTEN and SMAD7. *Clinical science* 129, 1237-1249 (2015).**
6. **Fang, L. et al. Circulating microRNAs as biomarkers for diffuse myocardial fibrosis in patients with hypertrophic cardiomyopathy. *Journal of translational medicine* 13, 314 (2015).**
7. **Cavarretta, E. & Condorelli, G. miR-21 and cardiac fibrosis: another brick in the wall? *European heart journal* 36, 2139-2141 (2015).**
8. **Selitsky, S.R. et al. Transcriptomic Analysis of Chronic Hepatitis B and C and Liver Cancer Reveals MicroRNA-Mediated Control of Cholesterol Synthesis Programs. *mBio* 6, e01500-01515 (2015).**
9. **Song, G. et al. MicroRNAs control hepatocyte proliferation during liver regeneration. *Hepatology* 51, 1735-1743 (2010).**
10. **Sheedy, F.J. et al. Negative regulation of TLR4 via targeting of the proinflammatory tumor suppressor PDCD4 by the microRNA miR-21. *Nature immunology* 11, 141-147 (2010).**
11. **Niu, J. et al. DNA damage induces NF-kappaB-dependent microRNA-21 up-regulation and promotes breast cancer cell invasion. *The Journal of biological chemistry* 287, 21783-21795 (2012).**
12. **Wu, H., Ng, R., Chen, X., Steer, C.J. & Song, G. MicroRNA-21 is a potential link between non-alcoholic fatty liver disease and hepatocellular carcinoma via modulation of the HBP1-p53-Srebp1c pathway. *Gut* (2015).**
13. **Damania, P. et al. Hepatitis B virus induces cell proliferation via HBx-induced microRNA-21 in hepatocellular carcinoma by targeting programmed cell death protein4 (PDCD4) and phosphatase and tensin homologue (PTEN). *PloS one* 9, e91745 (2014).**

14. Bandopadhyay, M. et al. Tumor suppressor micro RNA miR-145 and onco micro RNAs miR-21 and miR-222 expressions are differentially modulated by hepatitis B virus X protein in malignant hepatocytes. *BMC cancer* 14, 721 (2014).
15. Ladeiro, Y. et al. MicroRNA profiling in hepatocellular tumors is associated with clinical features and oncogene/tumor suppressor gene mutations. *Hepatology* 47, 1955-1963 (2008).
16. Chau, B.N. et al. MicroRNA-21 promotes fibrosis of the kidney by silencing metabolic pathways. *Science translational medicine* 4, 121ra118 (2012).
17. Saha, B., Bruneau, J.C., Kodys, K. & Szabo, G. Alcohol-induced miR-27a regulates differentiation and M2 macrophage polarization of normal human monocytes. *Journal of immunology* 194, 3079-3087 (2015).
18. Shirasaki, T. et al. MicroRNA-27a regulates lipid metabolism and inhibits hepatitis C virus replication in human hepatoma cells. *Journal of virology* 87, 5270-5286 (2013).
19. Singaravelu, R. et al. Hepatitis C virus induced up-regulation of microRNA-27: a novel mechanism for hepatic steatosis. *Hepatology* 59, 98-108 (2014).
20. Bandyopadhyay, S. et al. Hepatitis C virus infection and hepatic stellate cell activation downregulate miR-29: miR-29 overexpression reduces hepatitis C viral abundance in culture. *The Journal of infectious diseases* 203, 1753-1762 (2011).
21. Huang, Y.H. et al. Activation of Mir-29a in Activated Hepatic Stellate Cells Modulates Its Profibrogenic Phenotype through Inhibition of Histone Deacetylases 4. *PloS one* 10, e0136453 (2015).
22. Kong, G. et al. Upregulated microRNA-29a by hepatitis B virus X protein enhances hepatoma cell migration by targeting PTEN in cell culture model. *PloS one* 6, e19518 (2011).
23. Roderburg, C. et al. Micro-RNA profiling reveals a role for miR-29 in human and murine liver fibrosis. *Hepatology* 53, 209-218 (2011).
24. Tiao, M.M. et al. MicroRNA-29a protects against acute liver injury in a mouse model of obstructive jaundice via inhibition of the extrinsic apoptosis pathway. *Apoptosis : an international journal on programmed cell death* 19, 30-41 (2014).
25. Bihrer, V. et al. Serum miR-122 as a biomarker of necroinflammation in patients with chronic hepatitis C virus infection. *The American journal of gastroenterology* 106, 1663-1669 (2011).
26. Estrabaud, E. et al. Reduction of microRNA 122 expression in IFNL3 CT/TT carriers and during progression of fibrosis in patients with chronic hepatitis C. *Journal of virology* 88, 6394-6402 (2014).
27. Li, J. et al. miR-122 regulates collagen production via targeting hepatic stellate cells and suppressing P4HA1 expression. *Journal of hepatology* 58, 522-528 (2013).
28. Marquez, R.T. et al. Correlation between microRNA expression levels and clinical parameters associated with chronic hepatitis C viral infection in humans. *Laboratory investigation; a journal of technical methods and pathology* 90, 1727-1736 (2010).
29. Trebicka, J. et al. Hepatic and serum levels of miR-122 after chronic HCV-induced fibrosis. *Journal of hepatology* 58, 234-239 (2013).
30. Bala, S. et al. Increased microRNA-155 expression in the serum and peripheral monocytes in chronic HCV infection. *Journal of translational medicine* 10, 151 (2012).
31. Du, J. et al. MiR-146a-5p suppresses activation and proliferation of hepatic stellate cells in nonalcoholic fibrosing steatohepatitis through directly targeting Wnt1 and Wnt5a. *Scientific reports* 5, 16163 (2015).
32. Li, J.F. et al. Upregulation of microRNA-146a by hepatitis B virus X protein contributes to hepatitis development by downregulating complement factor H. *mBio* 6 (2015).
33. Motawi, T.K., Shaker, O.G., El-Maraghy, S.A. & Senousy, M.A. Serum MicroRNAs as Potential Biomarkers for Early Diagnosis of Hepatitis C Virus-Related Hepatocellular Carcinoma in Egyptian Patients. *PloS one* 10, e0137706 (2015).

34. Wang, B. et al. Role of microRNA-155 at early stages of hepatocarcinogenesis induced by choline-deficient and amino acid-defined diet in C57BL/6 mice. *Hepatology* 50, 1152-1161 (2009).
35. Bae, H.J. et al. MicroRNA-221 governs tumor suppressor HDAC6 to potentiate malignant progression of liver cancer. *Journal of hepatology* 63, 408-419 (2015).
36. Chen, J.J. et al. HBx protein-induced upregulation of microRNA-221 promotes aberrant proliferation in HBVrelated hepatocellular carcinoma by targeting estrogen receptor-alpha. *Oncology reports* 33, 792-798 (2015).
37. Ogawa, T. et al. MicroRNA-221/222 upregulation indicates the activation of stellate cells and the progression of liver fibrosis. *Gut* 61, 1600-1609 (2012).
38. Pineau, P. et al. miR-221 overexpression contributes to liver tumorigenesis. *Proceedings of the National Academy of Sciences of the United States of America* 107, 264-269 (2010).
39. Dong, R. et al. miR-222 overexpression may contribute to liver fibrosis in biliary atresia by targeting PPP2R2A. *Journal of pediatric gastroenterology and nutrition* 60, 84-90 (2015).
40. Shen, W.J., Dong, R., Chen, G. & Zheng, S. microRNA-222 modulates liver fibrosis in a murine model of biliary atresia. *Biochemical and biophysical research communications* 446, 155-159 (2014).
41. An, F. et al. MicroRNA-224 Induces G1/S Checkpoint Release in Liver Cancer. *Journal of clinical medicine* 4, 1713-1728 (2015).
42. Lin, L., Lu, B., Yu, J., Liu, W. & Zhou, A. Serum miR-224 as a biomarker for detection of hepatocellular carcinoma at early stage. *Clinics and research in hepatology and gastroenterology* (2015).
43. Scisciani, C. et al. Transcriptional regulation of miR-224 upregulated in human HCCs by NFkappaB inflammatory pathways. *Journal of hepatology* 56, 855-861 (2012).

Table S12: Characteristics of the patients with chronic hepatitis B and C selected for the selection of miRNAs and for whom liver samples are available.

	Chronic hepatitis B		Chronic hepatitis C	
	F0/F1/F2	F3/F4	F0/F1/F2	F3/F4
N, patients	12	12	12	12
Gender: male / female, n (%)	9 (75%)/ 3 (25%)	8 (66.6%)/ 4 (33.4%)	2 (16.7%) / 10 (83.3%)	7 (58.3%)/ 5 (41.7%)
Age, years (median, IQ)	47 (43.5-53.7)	41.5 (36.5-46.2)	48 (43.5-55.5)	47.5 (42.5-58.7)
BMI, $kg.m^{-2}$ (median, IQ)	24.9 (23.3-27.4)	27.8 (23.8-30.2)	26.8 (21.1-30.3)	26.5 (24.5-28.7)
ALT, IU/L (median, IQ)	63.5 (45.7-93)	56 (30-76.5)	94 (72.25-100.7)	122 (81.5-175)
AST, IU/L (median, IQ)	40 (31.7-58.2)	53 (35.25-63.25)	50 (42-83)	80 (61.2-89.7)
ALP, IU/L (median, IQ)	34 (23.75-49)	52 (36.5-120.75)	76.5 (64.2-90.2)	59 (57.7-107)
GGT, IU/L (median, IQ)	34 (31.75-58.25)	53 (36.5-120.8)	31 (26-46.5)	82 (56.7-137.7)
Platelets, $x10^3/mm^3$(median, IQ)	184.5 (158.7-229)	148.5 (96.7-209)	226.5 (187.7-272.2)	184 (140.5-212.7)
Cholesterol, mmol/L (median, IQ)			5.07-4.7-5.36)	3.96 (3.96-4.56)
Triglycerides, mmol/L (median, IQ)	0.83 (0.70-1.07)	1.14 (0.65-1.46)	0.7 (0.5-0.9)	0.9 (0.8-1.1)
Glycemia, mmol/L (median, IQ)	4.3 (4.1-4.7)	5.2 (4.7-6.7)	4.5 (4.4-4.9)	5.3 (4.6-5.6)
Total bilirubin, µmol/L (median, IQ)	16.5 (11.7-18.2)	18 (16.7-26.2)	13.5 (10.7-16)	12 (10.5-15.2)
Albumin, g/L (median, IQ)	46.2 (41.9-50.6)	42.4 (41.3-45.1)	46.4 (44-49.2)	45 (43.3-46)
Viral Loads, logUI/mL (median, IQ)	6.92 (5.65-7.62)	5.33 (2.36-6.42)	5.29 (4.85-5.67)	5.46 (4.76-6.20)
Necroinflammatory activity, n (%)				
None (A0)	0	1 (8.35%)	1 (8.35%)	1 (8.35%)
Mild (A1)	7 (58.3%)	8 (66.6%)	7 (58.3%)	6 (50%)
Moderate (A2)	5 (41.7%)	1 (8.35%)	3 (25%)	5 (41.35%)
Severe (A3)	0	2 (16.7%)	1 (8.35%)	0
NA	0	0	0	0
Steatosis Grades, n (%)				
0	6 (50%)	6 (50%)	8 (66.6%)	3 (25%)
1	4 (33.3%)	2 (16.65%)	2 (16.7%)	1 (8.35%)
2	1 (8.35%)	3 (25%)	2 (16.7%)	6 (50%)
3	1 (8.35%)	1 (8.35%)	0	2 (16.65%)
NA	0	0	0	0

HCV genotypes, n (%)	-	-		
1	-	-	5 (41.55%)	7 (58.3%)
2	-	-	2 (16.7%)	0
3	-	-	2 (16.7%)	1 (8.35%)
4	-	-	2 (16.7%)	4 (33.35%)
5	-	-	0	0
6	-	-	0	0
NA	-	-	1 (8.35%)	0

AST, aspartate aminotransferase; ALT, alanine aminotransferase; ALP, alkaline phosphate; BMI, Body mass index; GGT, Gamma-glutamyl transpeptidase, IQ: Interquartile range, NA: Not available.

Clinical parameters are expressed as median and interquartile range (IQ), unless indicated differentially. Differences between patients with F0-F1-F2 and those with F3-F4 were evaluated with the Fisher exact (qualitative variables) and the Wilcoxon rank-sum test (continuous variables).

Table S13: Characteristics of the patients with chronic hepatitis B and C selected for the selection of miRNAs and for whom serum samples are available.

	Chronic hepatitis B		Chronic hepatitis C	
	**F0/F1/F2**	**F3/F4**	**F0/F1/F2**	**F3/F4**
N, patients	12	12	12	12
Gender: male / female, n (%)	11 (92%)/1 (8%)	11 (92%)/1 (8%)	7 (58.3%)/ 5 (41.7%)	5 (41.7%) / 7(58.3%)
Age, years (median, IQ)	32.5 (29.5-43.5)	39.5 (32-46.7)	45.5 (41.7-49)	52 (45-56)
BMI, kg.m^{-2} (median, IQ)	25.12 (24.47-26.71)	25.82 (23.44-27.54)	23.31 (21.97-24.62)	30.98 (30.28-31.15)
ALT, IU/L (median, IQ)	72.5 (60-186.5)	105 (55-265.25)	84 (71-128)	69.5 (53.5-121.75)
AST, IU/L (median, IQ)	46.5 (38-90)	61 (34.75-115.25)	55 (41.75-75.5)	67 (46-104.25)
ALP, IU/L (median, IQ)	47.5 (35.2-60.2)	94 (44.5-229.5)	78.5 (43.85-90)	84 (69.5-103.5)
GGT, IU/L (median, IQ)	47.5 (35.25-60.25)	94 (44.5-229.5)	69.5 (52.75-157.25)	91 (77.5-115)
Platelets, x10^3/mm^3(median, IQ)	204.5 (182.5-226.5)	169.5 (150.75-196.25)	214.5 (203.25-255)	178 (137.5-196.5)
Cholesterol, mmol/L (median, IQ)	-	-	4.6 (4.12-4.71)	3.4 (3.29-3.85)
Triglycerides, mmol/L (median, IQ)	0.96 (0.71-1.24)	0.85 (0.63-1.24)	0.75 (0.61-0.84)	1.24 (0.68-1.53)
Glycemia, mmol/L (median, IQ)	5 (4.3-5.1)	4.5 (4.3-6.1)	4.8 (4.25-5.05)	4.93 (4.8-7.25)
Total bilirubin, µmol/L (median, IQ)	14 (11.04-19.5)	15 (16.5-30.5)	12 (10-14.25)	15 (12.5-16)

Albumin, g/L (median, IQ)	47.1 (43.2-47.75)	44 (41.9-45.9)	45.9 (44.6-46.9)	43.8 (41.9-45.1)
Viral Loads, logUI/mL (median, IQ)	7.14 (4.84-7.79)	6.42 (5.39-7.39)	5.96 (5.77-6.12)	5.58 (5.41-6.12)
Necroinflammatory activity, n (%)				
None (A0)	0	5 (41.7%)	0	0
Mild (A1)	8 (66.7%)	4 (33.3%)	9 (75%)	7 (58.35%)
Moderate (A2)	4 (33.3%)	3 (25%)	3(25%)	4 (33.3%)
Severe (A3)	0	0	0	1 (8.35%)
NA	0	0	0	0
Steatosis Grades, n (%)				
0	5 (41.7%)	6 (50%)	3 (25%)	3 (25%)
1	5 (41.7%)	2 (16%)	6 (50%)	3 (25%)
2	2 (16.6%)	4 (34%)	3 (25%)	5 (41.65%)
3	0	0	0	1 (8.35%)
NA	0	0	0	0
HCV genotypes, n (%)	-	-		
1	-	-	7 (58.3%)	6 (50%)
2	-	-	0	0
3	-	-	0	1 (8.35%)
4	-	-	2 (16.7%)	4 (33.3%)
5	-	-	0	0
6	-	-	0	0
NA	-	-	3 (25%)	1 (8.35%)

AST, aspartate aminotransferase; ALT, alanine aminotransferase; ALP, alkaline phosphate; BMI, Body mass index; GGT, Gamma-glutamyl transpeptidase, IQ: Interquartile range, NA: Not available.

Clinical parameters are expressed as median and interquartile range (IQ), unless indicated differentially. Differences between patients with F0-F1-F2 and those with F3-F4 were evaluated with the Fisher exact (qualitative variables) and the Wilcoxon rank-sum test (continuous variables).

Legends of the supplementary figures:

Supplementary figure 1: Differences in the expression of hepatic mir-27a, -27b, -29c and -155 according to the stage of fibrosis in patients with chronic hepatitis B and C. The expression of hepatic mir-27a, -27b, -29c and -155 was assessed by RT-qPCR from 1 ng of cDNA and compared in patients with F0-F1-F2 and F3-F4, in chronic hepatitis B patients (A-D) and in those with chronic hepatitis C (E-H). The ΔCt ($\Delta Ct=2^{\Delta Cp,\ sample}$) of each miRNA was calculated and normalized to the ΔCt value of SNORD44 in each biopsy. The log expression of the ratio miRNA/SNORD44 is shown as dot plot, each dot represents one patient (mean and standard deviation). The Wilcoxon rank-sum test was used to compare miRNAs expression.

Supplementary figure 2: Differences of the expression of serum mir-27a, -27b, -29c and -155 according to the stages of fibrosis in patients with chronc hepatitis B and C. The expression of serum mir-27a, -27b, -29c and -155 was assessed by RT-qPCR and compared in patients with F0-F1-F2 and F3-F3, in chronic hepatitis B patients (A-D) and in those with chronic hepatitis C (E-H). The ΔCt ($\Delta Ct=2^{\Delta Cp,\ sample}$) of each miRNA was calculated and normalized to the ΔCt value of the exogenous *C. elegans*-miR-39 (*cel*-miR-39) in each serum. The log expression of the ratio miRNA/*cel*-miR-39 is shown as dot plot, each dot represents one patient (mean and standard deviation). The Wilcoxon rank-sum test was used to compare miRNAs expression.

Supplementary Figure 3: **Variation of serum miRNAs expression after abrogation of viral replication in patients with chronic hepatitis B (A-E) and C (F-I). (A-E).** The expression of serum miR-29a, -92a, -122, -146a and -222 was compared in CHB patients with F0-F2 and F3-F4 before and 1 year after the initiation of NUcs. All the patients had undectectale HBV DNA at the second serum sample. (F-I) the expression of MiR-122, -221, -222 and -224 was compared in serum samples of CHC patients with F0-F1 and F3-F4, before and one year after the diagnosis of sustained virological response. The ΔCt ($\Delta Ct=2^{\Delta Cp,\ sample}$) of each miRNA was calculated and normalized to the ΔCt value of the exogenous *C. elegans*-miR-39 (*cel*-miR-39) in each serum. The log expression of the ratio miRNA/*cel*-miR-39 is shown as dot plot, each dot represents one patient (mean and standard

deviation). The Wilcoxon rank-sum test was used to compare miRNAs expression.

Supplementary figure 4: Comparison of the expression of serum and hepatic mir-27a, -27b, -29c and -155 in patients with chronic hepatitis B and C. The expression of mir-27a, -27b, -29c and -155 was assessed by RT-qPCR and compared in serum (A-D) and liver (E-H) samples of patients with CHB and CHC. (A-D) In serum samples, The ΔCt ($\Delta Ct=2^{\Delta Cp,\ sample}$) of each miRNA was calculated and normalized to the ΔCt value of the exogenous *C. elegans*-miR-39 (*cel*-miR-39) in each serum. The log expression of the ratio miRNA/*cel*-miR-39 is shown as dot plot, each dot represents one patient (mean and standard deviation). (E-H) In liver samples, the ΔCt ($\Delta Ct=2^{\Delta Cp,\ sample}$) of each miRNA was calculated and normalized to the ΔCt value of SNORD44 in each biopsy. The log expression of the ratio miRNA/SNORD44 is shown as dot plot, each dot represents one patient (mean and standard deviation). The log expression of the ratio miRNA/SNORD44 is shown as dot plot, each dot represents one patient (mean and standard deviation). The Wilcoxon rank-sum test was used to compare miRNAs expression.

Supplementary figure 5: Differences in the expression of hepatic miR-26a, -26b, -199a/b-3p and -223 according to the stage of fibrosis in patients with chronic hepatitis B and C. The expression of hepatic miR-26a, -26b, -199a/b-3p and -223 was assessed by RT-qPCR from 1 ng of cDNA and compared in patients with F3-F4 and F0-F1-F2, in chronic hepatitis B patients (A-I) and in those with chronic hepatitis C (J to R). The ΔCt ($\Delta Ct=2^{\Delta Cp,\ sample}$) of each miRNA was calculated and normalized to the ΔCt value of SNORD44 in each biopsy. The log expression of the ratio miRNA/SNORD44 is shown as dot plot, each dot represents one patient (mean and standard deviation). The Wilcoxon rank-sum test was used to compare miRNAs expression.

Supplementary figure 6: Differences of the expression of serum miR-26a, -26b, -199a/b-3p and -223 according to the stages of fibrosis in patients with chronic hepatitis B and C. The expression of serum miR-26a, -26b, -199a/b-3p and -223 was assessed by RT-qPCR and compared in patients with F3-F4 and F0-F1-F2, in chronic hepatitis B patients (A-I) and in those with chronic hepatitis C (J to R). The ΔCt ($\Delta Ct=2^{\Delta Cp,\ sample}$) of each miRNA was calculated and normalized to the ΔCt value of the

exogenous *C. elegans*-miR-39 (*cel*-miR-39) in each serum. The log expression of the ratio miRNA/*cel*-miR-39 is shown as dot plot, each dot represents one patient (mean and standard deviation). The Wilcoxon rank-sum test was used to compare miRNAs expression.

Supplementary figure 1

Chronic hepatitis B

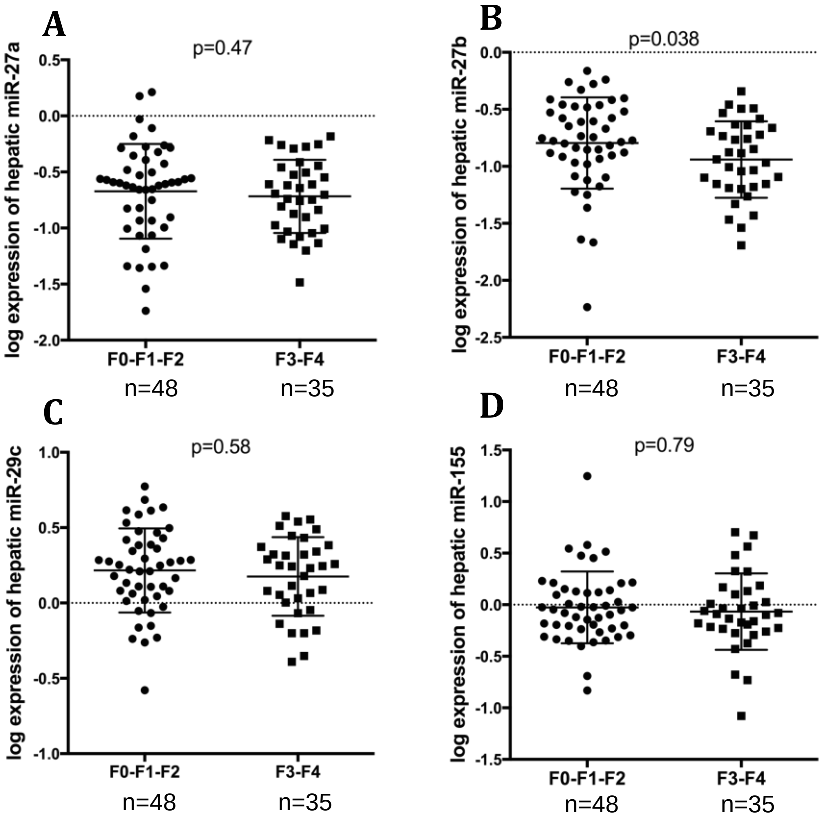

Chronic hepatitis C

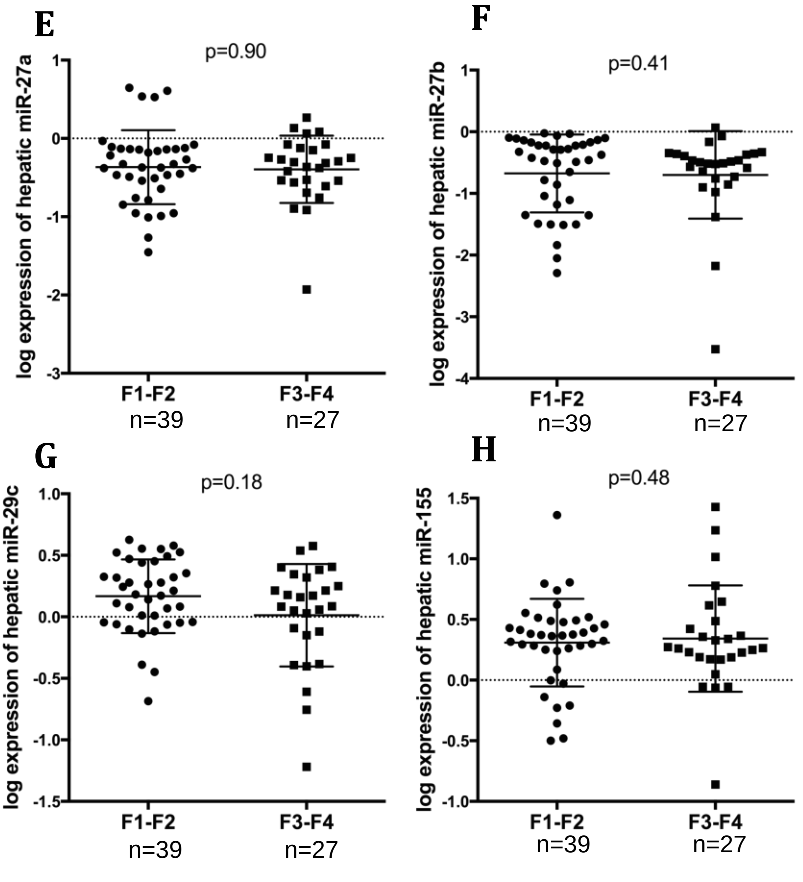

Supplementary figure 2

Chronic hepatitis B

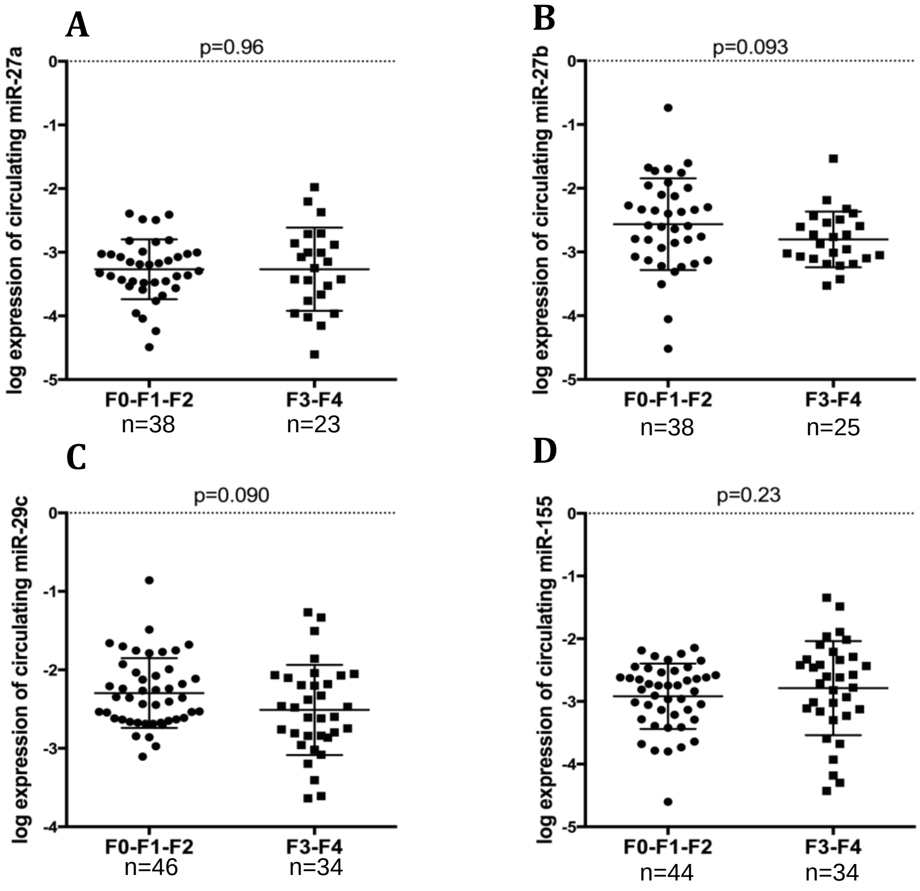

Supplementary figure 3

CHB - Before / After treatment

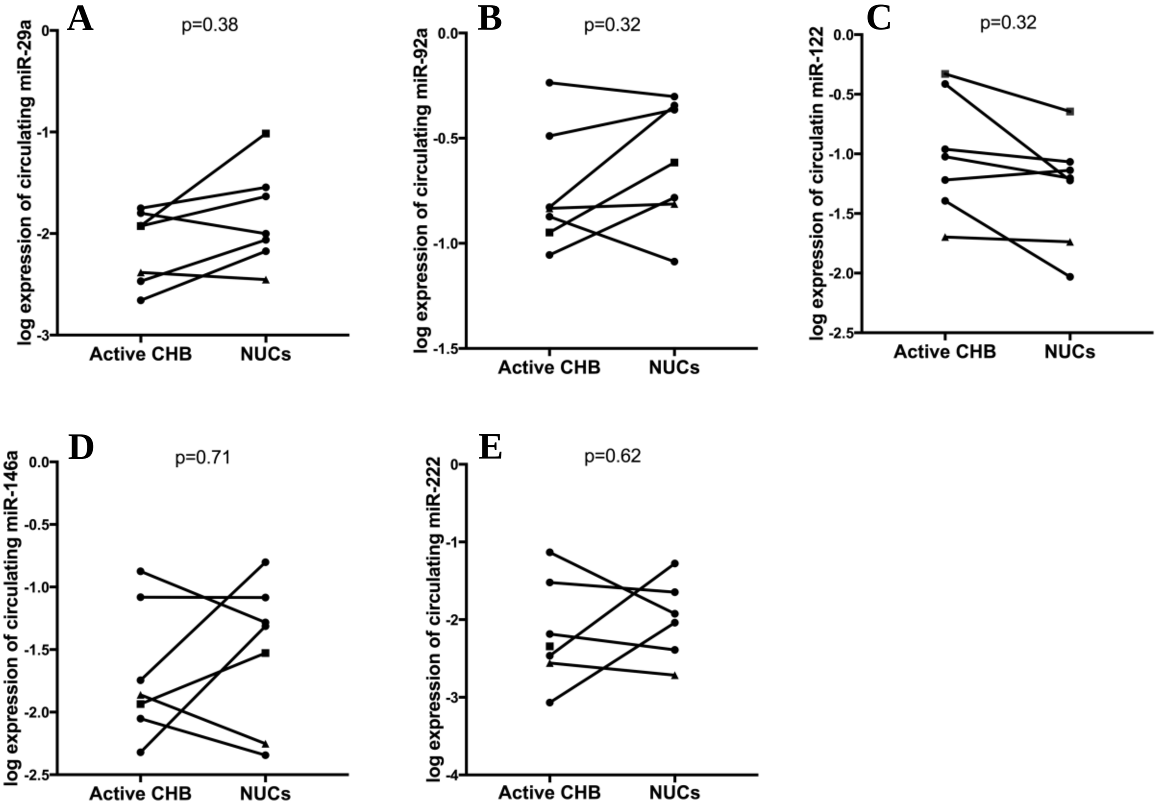

CHC - Before / After treatment

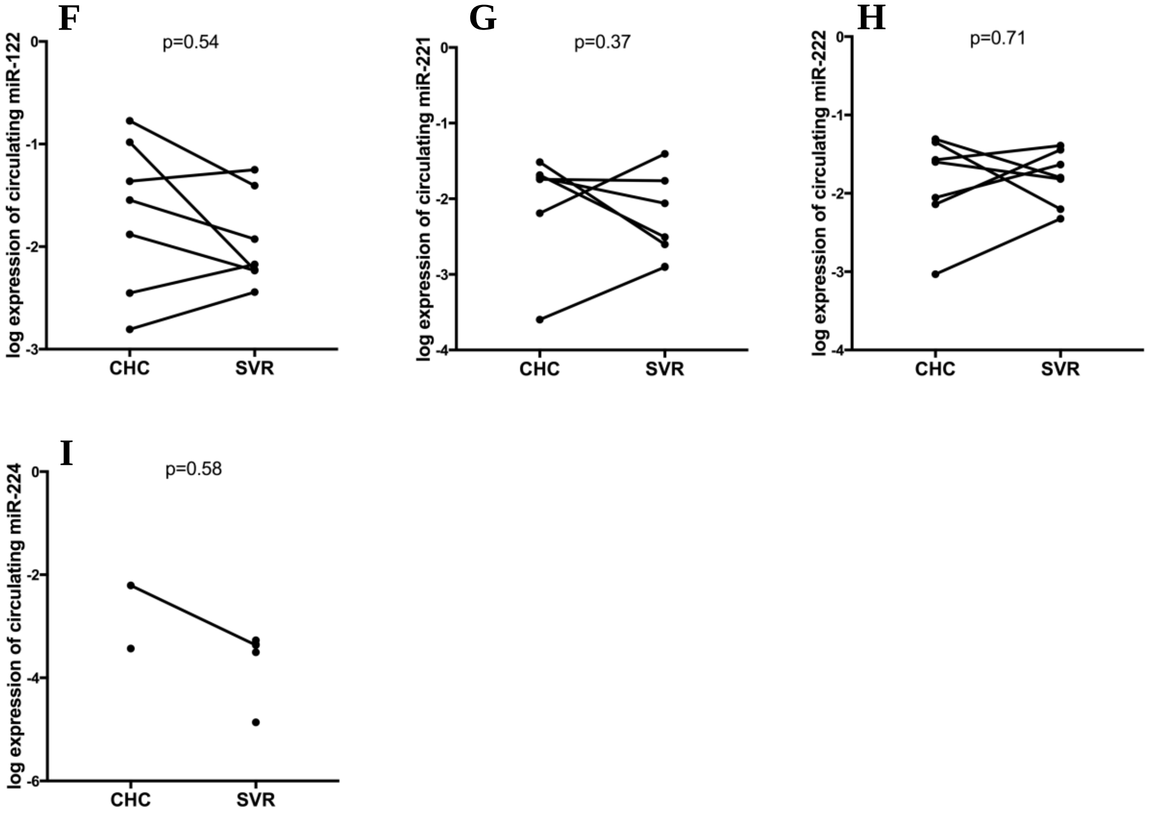

Supplementary figure 4

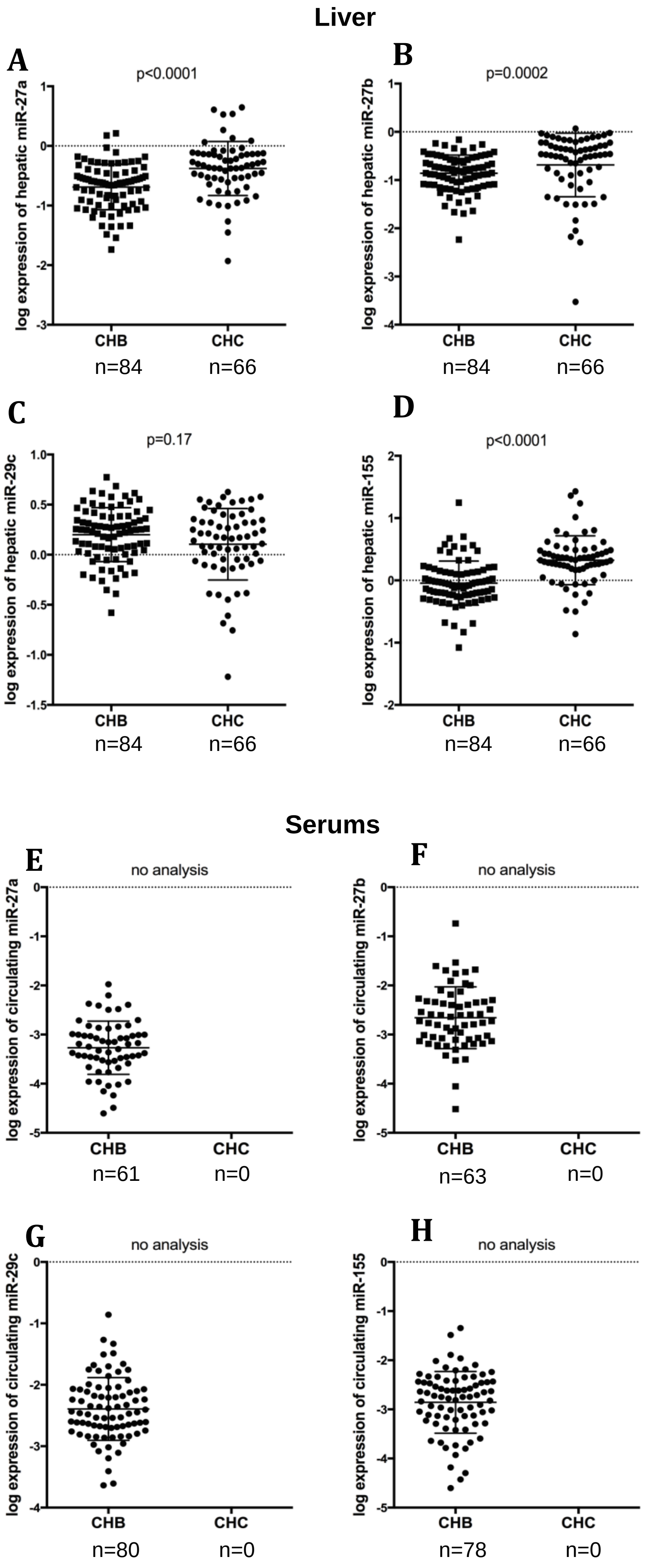

Supplementary figure 5

CHB - Biopsies

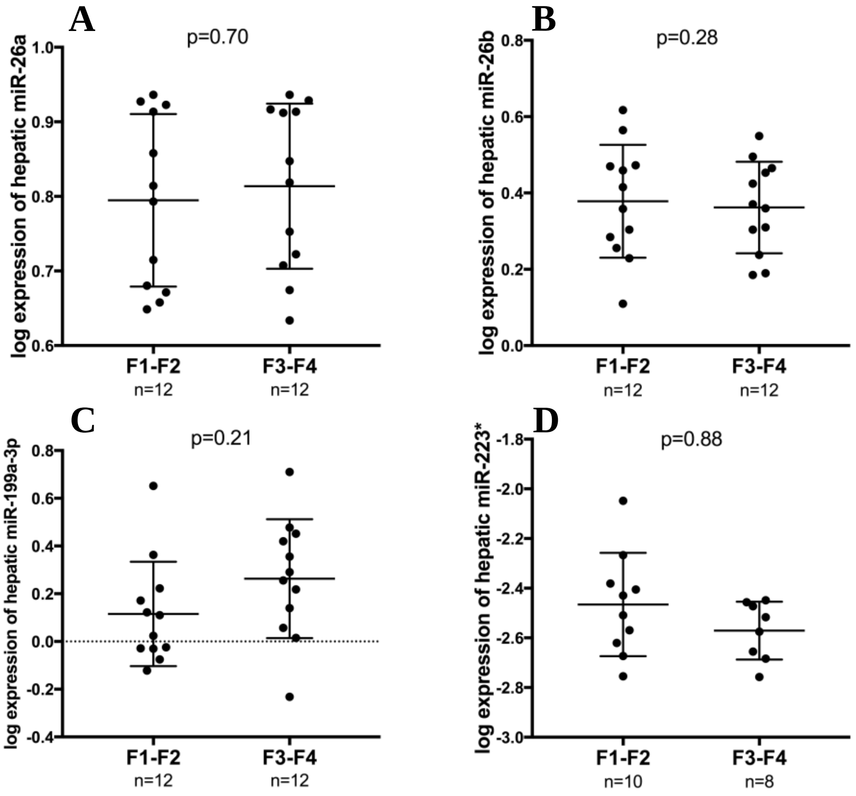

CHC - Biopsies

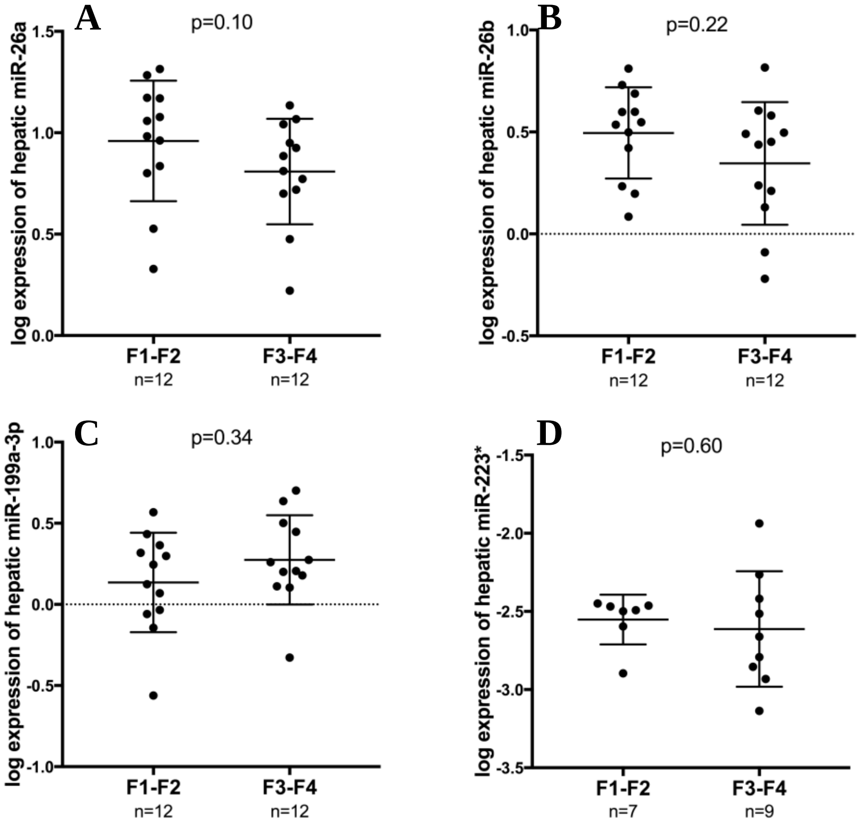

Supplementary figure 6

CHB - Serums

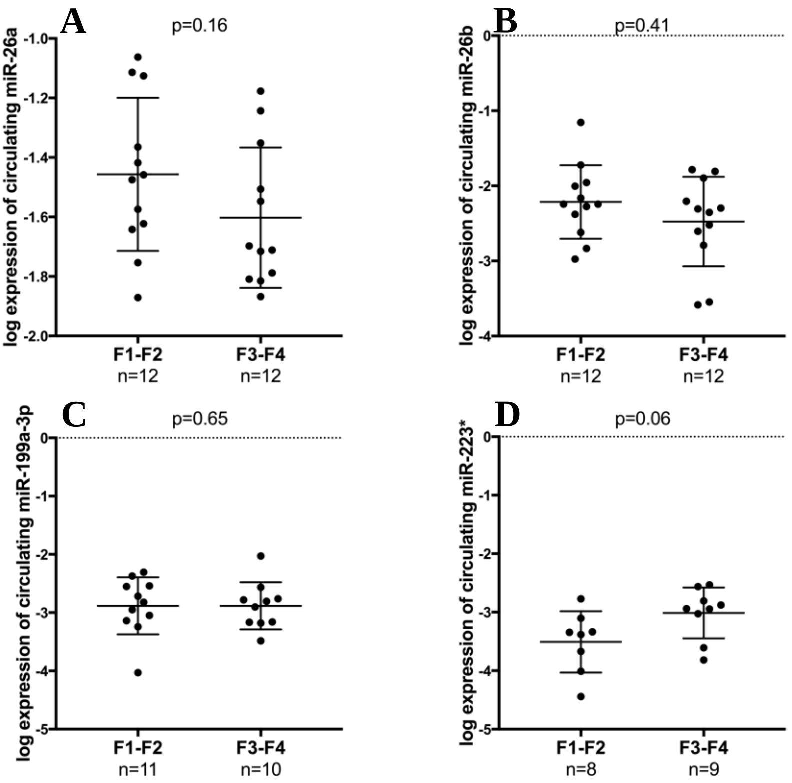

CHC - Serums

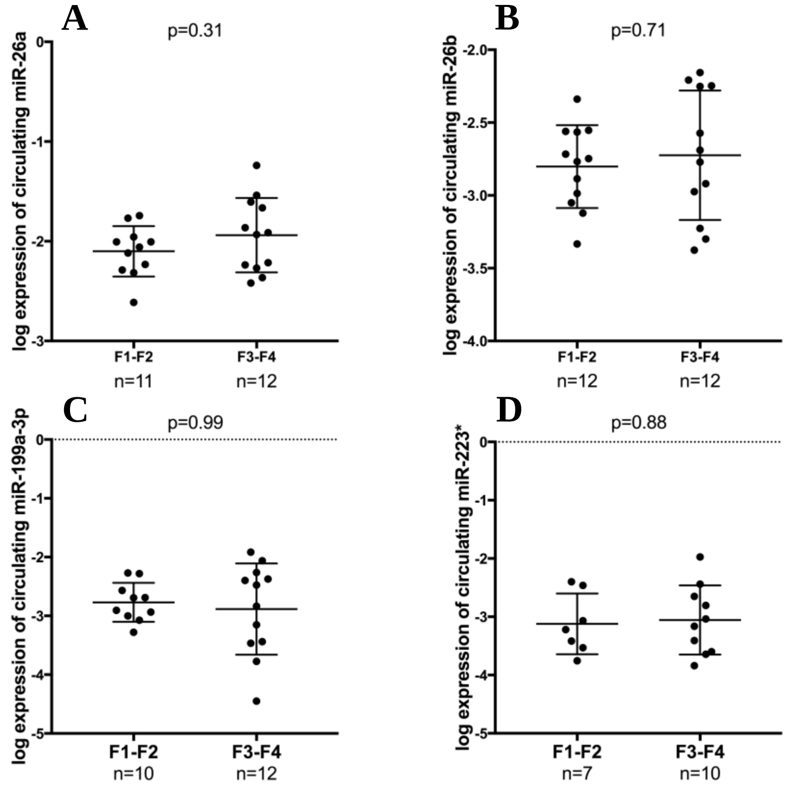

Supplement: Supplementary Information [file srep34935-s1.pdf]
